# Integrated multi-omics characterization of KRAS mutant colorectal cancer

**DOI:** 10.7150/thno.73089

**Published:** 2022-07-04

**Authors:** Wei Chong, Xingyu Zhu, Huicheng Ren, Chunshui Ye, Kang Xu, Zhe Wang, Shengtao Jia, Liang Shang, Leping Li, Hao Chen

**Affiliations:** 1 Department of Gastrointestinal Surgery, Shandong Provincial Hospital Affiliated to Shandong First Medical University, Jinan, China.; 2 Department of Gastrointestinal Surgery, Shandong Provincial Hospital, Cheeloo College of Medicine, Shandong University, Jinan, China.; 3 Key Laboratory of Engineering of Shandong Province, Shandong Provincial Hospital, Jinan, China.; 4 Medical Science and Technology Innovation Center, Shandong First Medical University & Shandong Academy of Medical Sciences, Jinan, China.; 5 Department of Tumor Cell Biology, National Clinical Research Center for Cancer, Tianjin's Clinical Research Center for Cancer, Tianjin Medical University Cancer Institute and Hospital, Tianjin, China.; 6 Clinical Research Center of Shandong University, Clinical Epidemiology Unit, Qilu Hospital of Shandong University, Jinan, Shandong, 250021, China.

**Keywords:** Colorectal cancer, KRAS mutation, Proteogenomics, Molecular subtype, Prognosis

## Abstract

KRAS mutation is the most frequent oncogenic aberration in colorectal cancer (CRC). The molecular mechanism and clinical implications of KRAS mutation in CRC remain unclear and show high heterogeneity within these tumors.

**Methods:** We harnessed the multi-omics data (genomic, transcriptomic, proteomic, and phosphoproteomic etc.) of KRAS-mutant CRC tumors and performed unsupervised clustering to identify proteomics-based subgroups and molecular characterization.

**Results:** In-depth analysis of the tumor microenvironment by single-cell transcriptomic revealed the cellular landscape of KRAS-mutant CRC tumors and identified the specific cell subsets with KRAS mutation. Integrated multi-omics analyses separated the KRAS-mutant tumors into two distinct molecular subtypes, termed KRAS-M1 (KM1) and KRAS-M2 (KM2). The two subtypes had a similar distribution of mutated residues in KRAS (G12D/V/C etc.) but were characterized by distinct features in terms of prognosis, genetic alterations, microenvironment dysregulation, biological phenotype, and potential therapeutic approaches. Proteogenomic analyses revealed that the EMT, TGF-β and angiogenesis pathways were enriched in the KM2 subtype and that the KM2 subtype was associated with the mesenchymal phenotype-related CMS4 subtype, which indicated stromal invasion and worse prognosis. The KM1 subtype was characterized predominantly by activation of the cell cycle, E2F and RNA transcription and was associated with the chromosomal instability (CIN)-related ProS-E proteomic subtype, which suggested cyclin-dependent features and better survival outcomes. Moreover, drug sensitivity analyses based on three compound databases revealed subgroup-specific agents for KM1 and KM2 tumors.

**Conclusions:** This study clarifies the molecular heterogeneity of KRAS-mutant CRC and reveals new biological subtypes and therapeutic possibilities for these tumors.

## Introduction

Colorectal cancer (CRC) ranks third in terms of new cases and represents the second leading cause of cancer-related death worldwide 1. CRC is widely considered a heterogeneous disease, with multiple gene alterations and numerous pathways involved in its pathogenesis 2. Kirsten rat sarcoma (KRAS) is one of the most frequently mutated oncogenes in CRC, with approximately 40% of CRC patients harboring activating missense mutations in KRAS, most of which occur at codons 12, 13 and 61 3. The KRAS gene encodes a guanosine triphosphate (GTP)/guanosine diphosphate (GDP)-binding protein that belongs to the guanosine triphosphatase (GTPase) RAS family and triggers a diverse range of phosphorylation cascades, including the canonical RAF/MEK/ERK, PI3K/AKT, and RALGDS/RAL pathways 4, 5. Once KRAS mutations occur, the hydrolysis of GTP is disrupted and/or nucleotide exchange is enhanced, leading to accumulation of KRAS in the active state and contributing to constitutive stimulation of downstream signaling pathways, thereby promoting tumor cell proliferation and survival. It has become more evident that oncogenic KRAS mutations mediate the tumor microenvironment (TME), particularly by promoting inflammation and suppressing the immune response and ultimately leading to immune evasion and tumor progression 6-8. However, the landscape of cellular heterogeneity in the TME has not been well characterized in CRCs with KRAS mutations and remains to be investigated. The complexity of the signaling network and the heterogeneous features of the multiple KRAS-mutant alleles have contributed to the difficulty in developing molecular targeted therapies against KRAS-mutant tumors.

Recently, allele-specific covalent inhibitors that can specifically bind to the cysteine residue in the KRAS-G12C mutant have shown promising outcomes in the clinic 9. However, clinical studies have unexpectedly reported that the response rate to these drugs is high in patients with non-small cell lung cancer (NSCLC) but limited in patients with colorectal cancer 10-12, suggesting the intertumor heterogeneity in KRAS-mutant cancers. Moreover, different mutations in KRAS residues may modulate the intrinsic activation (GTP-bound state) of the mutant protein and its interaction with downstream effectors 13. Ihle et al*.* reported that the KRAS-G12V and KRAS-G12C mutants preferentially activate RTK and RAL signaling and decrease AKT activation, whereas the KRAS-G12D mutant is prone to activating PI3K and MEK signaling 14. These findings indicate that KRAS-mutant tumors are still highly heterogeneous, and further exploration of biological subtypes and molecular targets is warranted to guide the prognosis and treatment of patients with KRAS-mutant CRC.

Previous large-scale omics studies on KRAS-mutant cancers have greatly advanced our understanding of the molecular diversity of KRAS-driven cancer 6, 15, 16. Thus far, the main efforts are based largely on genomics and transcriptomics. Of note, recent studies have demonstrated the importance of proteomic and phosphoproteomic characterization in advancing the understanding of tumor heterogeneity 17, 18. In addition to reinforcing or complementing transcriptomic data, integration of proteogenomic data may also correct inaccurate transcriptomic data-based inferences and lead to unexpected discoveries and therapeutic opportunities. Given that the phosphorylation cascade dominates KRAS-induced downstream signaling, obtaining a comprehensive profile of the proteome and phosphoproteome will be particularly informative to improve our understanding of the phenotypic heterogeneity of KRAS-mutant cancers 19, 20. In addition, the quantitative proteomics of a portion of the Cancer Cell Line Encyclopedia (CCLE) reported the association between gene expression and the growth dependency on these genes 21. A recent study characterized the cellular proteomic and phosphoproteomic landscapes of KRAS-mutant tumor cell lines from a combined set of lung, pancreatic adenocarcinoma, and colorectal tumors and identified the therapeutic potential of specific cell subsets 22. However, an integrative large-scale proteomic and phosphoproteomic analysis of patients with KRAS-mutant CRC is still lacking.

In this study, we integrated multi-omics (genomic, transcriptomic, proteomic, phosphoproteomic, etc*.*) data to comprehensively characterize the molecular network and biological heterogeneity and to refine the molecular stratification of KRAS-mutant CRC, which may facilitate the development of combination therapies that are more suitable for KRAS-mutant CRC. Here, we report that KRAS-mutated CRC tumors can be divided into two distinct molecular subtypes based on integrated transcriptomic, proteomic and phosphoproteomic profiling but not on the mutated residues. The two subtypes were characterized by different clinical features, biological pathways, copy number alterations, and phosphorylation cascades associated with KRAS signaling activation. Kinase network analysis and drug sensitivity prediction also revealed potential therapeutic agents that may be helpful to treat the specific subtype. Overall, these proteogenomic analyses present new avenues for biological discoveries and therapeutic development in KRAS-mutant CRC tumors.

## Materials and methods

### Curation and Preprocessing of Publicly Available Datasets

Multi-omics sequencing data and clinical annotations of colorectal carcinoma samples with detected KRAS mutations were retrospectively collected from publicly available datasets in the Clinical Proteomic Tumor Analysis Consortium (CPTAC) colon cancer database 17, The Cancer Genome Atlas-Colon Adenocarcinoma/Rectal Adenocarcinoma (TCGA-COAD/READ) cohort in cBioPortal, and the Chinese Colorectal Cancer (CCRC) cohort 23. Moreover, KRAS mutation sequenced by targeted MSK-IMPACT panel sequencing (MSK cohort) 3 and allelic discrimination by TaqMan probe sequencing (CIT[GSE39582] 24 and GSE87211 25 cohort) were employed to validate the clinical outcomes and molecular subtyping results. Finally, a total of 2579 CRC samples with available omics and clinical data were enrolled in this analysis (Table S1). The proteomic and phosphoproteomic data were mainly derived from the CPTAC and CCRC cohorts and are described in the supplementary files. The clinical information and subtype clustering of collected cohorts are provided in Table S2.

### Comparison of genomic alterations

The somatic mutation and copy number alteration (SCNA) segment data from the CPTAC cohort and TCGA-COAD/READ cohort were downloaded and curated for genomic analysis. Tumors with nonsynonymous mutations (including frameshift mutations, in-frame mutations, missense mutations, nonsense mutations and splice site mutations) in KRAS were considered KRAS-mutant tumors. The significantly mutated genes (SMGs) were curated from previous studies 26, 27 and plotted with the waterfall function in the 'GenVisR' R package. We adopted the 'maftools' package to extract the mutation signatures from the somatic mutation data (supplementary files). Next, GISTIC2 (version 2.0.23) 28 was used to retrieve gene-level copy number values and call significant copy number alterations in the cohort. The aneuploidy scores of TCGA samples were determined and curated from previous studies 29.

### Consensus Molecular Clustering for KRAS-Mutant Tumor Samples

We adopted three types of omics data—mRNA transcriptome data, imputed proteomic data and imputed phosphoproteomic data (the data imputation procedure is described in the supplementary files), to a similarity matrix using the R package “CancerSubtypes” 15, 30 with default parameters. The similarity matrix was used as the input for unsupervised clustering performed with the R package “ConsensusClusterPlus” 31. Variable selection analysis was used for gene signature selection of KRAS-mutant subset and was performed with the random forest algorithm.

### Inference of infiltrating cells in the TME of CRC

We utilized the xCell algorithm 32 to infer infiltrating immune and stromal cell subpopulations in CRC tumors based on bulk RNA-seq datasets. Gene expression profiles were prepared using standard annotation files, and data were uploaded to the xCell web portal (https://xcell.ucsf.edu/), with the algorithm run using the xCell signature.

### Single-cell analyses of the cellular landscape in CRC

Single-cell RNA-seq and metadata were curated from the Samsung Medical Center (SMC cohort) 33 and Katholieke Universiteit Leuven (KUL cohort) 34 and are available in the NCBI Gene Expression Omnibus (GEO) database under accession codes GSE132465 and GSE144735. The scRNA-seq analysis pipeline is summarized in the supplementary files.

### Colorectal cancer cell line and drug sensitivity analyses

Available clinical annotations and expression profiles of human colorectal cancer cell lines (N=112) were obtained from the Broad Institute Cancer Cell Line Encyclopedia (CCLE) project (https://portals.broadinstitute.org/ccle/) 35. Three drug sensitivity databases (CTRPv2.0, PRISM, and GDSC1) were accessed via the Cancer Dependency Map (DepMap) portal (https://depmap.org/portal/).

### Statistical analyses

The statistical analyses in this study were performed with R version 4.0.3. For quantitative data, statistical significance for normally distributed variables was estimated by Student's t test, and nonnormally distributed variables were analyzed by the Wilcoxon rank-sum test. For comparisons among more than two groups, the Kruskal-Wallis test and one-way analysis of variance were used as nonparametric and parametric methods, respectively. The chi-square test and Fisher's exact test were used to analyze contingency tables based on the specific grouping condition. Kaplan-Meier survival analysis and a Cox proportional hazards model were used to analyze the association between the TME modification pattern and prognosis. All comparisons were two-sided with an alpha level of 0.05, and the Benjamini-Hochberg method was applied for multiple hypothesis testing to control the false discovery rate (FDR).

## Results

### Molecular comparison of KRAS-mutant versus KRAS-wild-type colorectal cancer

**Figure 1:**
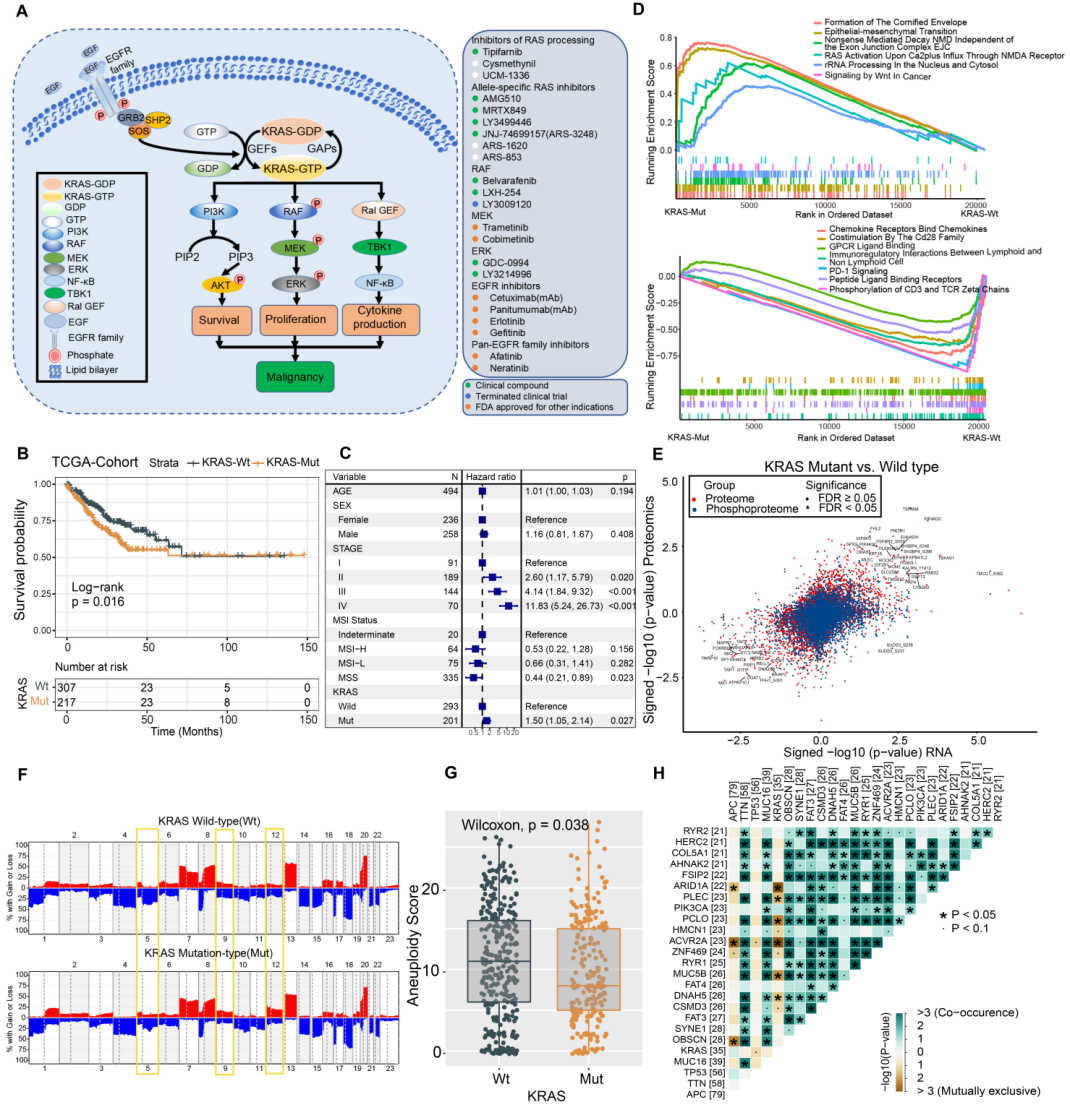
** Molecular Comparison on KRAS-Mut versus-wild type colorectal cancer.** (A) A schematic plot summarized the KRAS signaling pathway and relevant inhibitors of each node. (B) Kaplan-Meier curves for patients with KRAS-Mut and KRAS-WT groups in the TCGA-COAD/READ cohort. (C) Subgroup analysis estimating clinical prognostic value between KRAS-WT and KRAS-Mut type in TCGA cohort and cancer stage by univariate Cox regression. The length of the horizontal line represented the 95% confidence interval for each group. The vertical dotted line represented the hazard ratio (HR) of all patients. (D) Top enriched gene pathways with RNA expression profile in KRAS-Mut and -WT subgroups from TCGA cohort as assessed by using the GSEA algorithm. (E) Scatterplots showing significance of RNA, protein (red), phosphorylation site (blue) (signed -log10 p-value) abundance changes between KRAS-Mut and WT tumors in CPTAC cohort as determined using the Wilcoxon rank-sum test. All identified sites are represented and statistically significant gene (FDR < 0.05) specified by triangles. (F) Arm-level somatic copy-number alteration (SCNA) events in KRAS-Mut versus -WT. Red denotes amplification and blue denotes deletion. (G) Comparison of the aneuploidy score between KRAS-Mut and WT subtype in TCGA. (H) The relationships among the top 25 mutated genes (including KRAS) with different somatic mutations frequency.

We summarized the KRAS activation signaling pathway and relevant inhibitors of each node (Figure 1A). Once KRAS is mutated, the intrinsic GTP-GDP cycling in KRAS is disrupted, allowing the mutant KRAS protein to accumulate in an active state and thereby constitutively activate downstream MAPK and PI3K signaling cascades, which results in cell proliferation and survival. The various KRAS inhibitors listed in the box were developed to target each node of the KRAS signaling pathway and then evaluated in preclinical or clinical studies (Figure 1A). The lollipop plot shows that the most common KRAS mutations were KRAS-G12D/V/S/C, followed by G13D/C in the TCGA and CPTAC datasets (Figure S1A-S1B). We conducted survival analysis to investigate the prognostic value of KRAS mutation in CRC patients. Kaplan-Meier analysis indicated worse outcomes in the KRAS-mutant groups identified by whole-exome sequencing (TCGA cohort: HR, 1.46 [95% CI, 1.03 to 2.08], P = 0.016; Figure 1B, Table S3), and the association remained significant in the multivariable regression model after adjusting for age, sex, clinical stage, and MSI status (HR, 1.50 [95% CI, 1.05 to 2.14], P = 0.027; Figure 1C). We also validated the results in independent targeted panel sequencing cohorts (CIT cohort: HR, 1.42 [95% CI, 1.03 to 1.95], P = 0.023; MSK cohort: HR, 1.32 [95% CI, 1.16 to 1.49], P = 0.011; Figure S1C). To obtain mechanistic insights into the different clinical outcomes in the KRAS-mutant (Mut) and KRAS-wild-type (WT) groups, we carried out gene set enrichment analysis (GSEA) based on transcriptomic expression profiles. The KRAS-Mut group exhibited higher enrichment in oncogenic signaling pathways, such as epithelial-mesenchymal transition and Wnt signaling in tumors (Figure 1D, upper panel). However, the KRAS-WT group exhibited enrichment mainly in the gene sets of chemokine receptors bind chemokines, immunoregulatory interactions between a lymphoid and a nonlymphoid cell, PD-1 signaling, etc., indicating stronger immune activation (Figure 1D, lower panel). We also utilized proteomic data to identify the differentially regulated proteins and overrepresented pathways. Tumor malignant phenotype related IGFBP2 and KRT18 were significantly enriched in KRAS-Mut subtype, whereas neutrophils and macrophages related CD177, MMP1 and ARG1 were enriched in WT subtypes (Figure S1D-S1E). Next, we curated transcriptomic, proteomic and phosphoproteomic data from the CPTAC cohort and compared the global differential regulation in KRAS-Mut versus KRAS-WT tumors. KRAS-Mut tumors showed significant upregulation of tumor migration (TGFBR2-S553 and EPHB3) and PI3K/AKT activation (PIP4K2C and PIK3R1), while the KRAS-WT group was enriched in immune regulation (TAP1/2, IFITM1, and IFIH1-S301) and cellular metabolism (DGAT1 and HINT3) (Figure 1E, Table S4).

Accumulating evidence has focused on the genomic mutations that are implicated in tumorigenesis and immune evasion and confer selective advantages during the evolution of cancer. We first compared the tumor mutation load in KRAS-Mut versus KRAS-WT and observed that there was no difference in the TCGA and CPTAC cohorts (Figure S1F-S1G). Immune response ratio in most types of tumors who received immune checkpoint inhibitors was not significantly different between the KRAS-Mut and -WT groups (Figure S1H). We further compared the somatic copy number alterations (SCNAs) and aneuploidy scores in KRAS-Mut and KRAS-WT samples. At the chromosomal level, KRAS-Mut tumors showed a lower degree of arm-level SCNAs than WT tumors, and the alterations were mainly concentrated on chr5q, chr9q and chr12 (Figure 1F). Moreover, we observed significantly lower aneuploidy scores (total number of arm-level gains and losses in a tumor) in KRAS-Mut versus WT tumors (P = 0.038, Figure 1G). Using the somatic interactions function, we performed a pairwise Fisher's exact test to detect the relationships between KRAS mutation and the 25 most commonly mutated genes in CRC (Figure 1H). ARID1A and ACVR2A mutations were mutually exclusive with KRAS mutations in tumors, further indicating the genetic aberrations in CRC.

### Profiling the single-cell transcriptomic landscape of KRAS-Mut colorectal cancer

**Figure 2:**
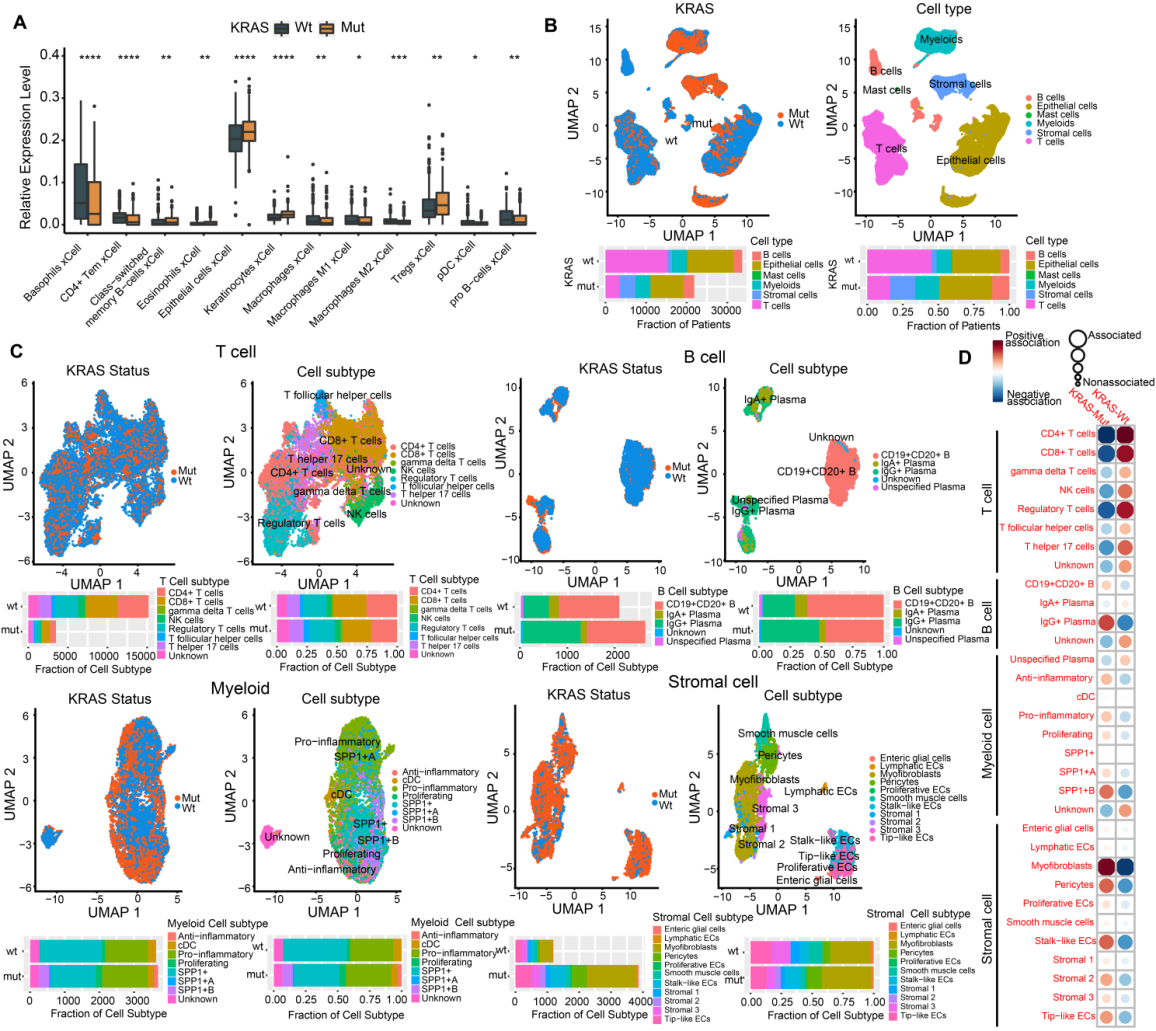
** Profiling single-cell transcriptomes landscape of KRAS-Mut colorectal cancer.** (A) Comparison of xCell algorithm inferred cell infiltration level based on bulk RNA-seq between the KRAS-Mut and WT tumors. Within each group, the thick line represents the median value. The bottom and top of the boxes are the 25th and 75th percentiles (interquartile range). The whiskers encompass 1.5 times the interquartile range. The range of P values are labeled above each boxplot with asterisks (*P < 0.05, **P < 0.01, ***P < 0.001). (B) UMAP plots showing the 55539 colorectal cancer cells derived from scRNA-seq and color-coded by KRAS-Mut and WT (left panel); The CRC single cells were clustered into six major cell types (right panel). Bar plot represented the cell population counts (left bottom) and proportions (right bottom) in KRAS-Mut and WT colorectal cancer tissues. (C) Cell subpopulations of T cells, B cells, Myeloid cells, and stromal cells in KRAS-Mut and WT tumors with color-coded by cell subtype and cluster numbers (Upper). Bar plot of cell counts and proportions in KRAS-Mut and WT colorectal cancer tissues (Lower). (D) Alterations of cellular subpopulations dynamics between KRAS-Mut and WT tumors are shown. Dot size represents Pearson's residual of the chi-squared test and the color represents the degree of positive or negative association from Pearson's residual of the chi-squared test.

Numerous studies have shown that the TME plays a crucial role in tumor progression and immune escape and has an effect on the response to immunotherapy^36^, ^37^. Here, we utilized the xCell algorithm based on bulk RNA-seq data to quantify stromal and immune cell infiltration, and investigated the variation of cell subpopulations in CRC. Most of the cell subpopulations were significantly different between the KRAS-Mut and KRAS-WT tumors. Tumors with KRAS mutation were characterized by increases in epithelial cells and Treg cells, whereas KRAS-WT tumors were distinguished by CD4^+^ T cells, macrophages, pDCs, etc. (Figure 2A).

To delineate the cell subpopulation landscape of KRAS-Mut CRC more accurately, we collected and curated 10x single-cell RNA-seq transcriptome data from the SMC and KUL datasets and integrated them into a combined scRNA-seq dataset via a reciprocal PCA workflow (Methods section, Figure S2A). Finally, a total of 55539 single cells derived from 16 KRAS-WT and 13 KRAS-Mut colorectal tumors were obtained and analyzed by Seurat 4.0 (Figure 2B, left panel). We adopted a graph-based clustering approach (K-nearest neighbor) based on the Euclidean distance in PCA space and performed modularity optimization with the Louvain algorithm to cluster the CRC cells into 29 clusters with differential expressed features (Figure S2A-S2B). Via marker-based annotations from Lee et al. 33, 38, six major cell types were identified, namely, lymphocytes/plasma cells, epithelial cells, fibroblasts/endothelial cells, myeloid cells, T/natural killer (NK)/NK T lymphocytes, and mast cells (Figure 2B, right panel). We found that T cells, B cells, myeloid cells, and stromal cells were significantly differentially distributed in KRAS-Mut versus KRAS-WT tumors based on the chi-square test (Figure 2B, right panel). Therefore, we extracted the four major cell types and further subdivided them into specific cell subpopulations. We employed the same workflow described above and classified the T cells, B cells, myeloid cells, and stromal cells into eight, five, eight, and eleven subpopulations, respectively (Figure 2C). Comparative analyses showed that the cell subcluster distributions of CD4^+^ T cells, CD8^+^ T cells and regulatory T cells were significantly elevated in KRAS-WT tumors, whereas IgG^+^ plasma cells (B cells), SPP1^+^ macrophages (myeloid cells) and myofibroblasts (stromal cells) were significantly increased in KRAS-Mut tumors (Figure 2C-2D). Representative markers of these key cell subclusters on the UMAP plot are shown in Figure S2C.

### Identifying the molecular subtypes of KRAS-Mut subsets

**Figure 3:**
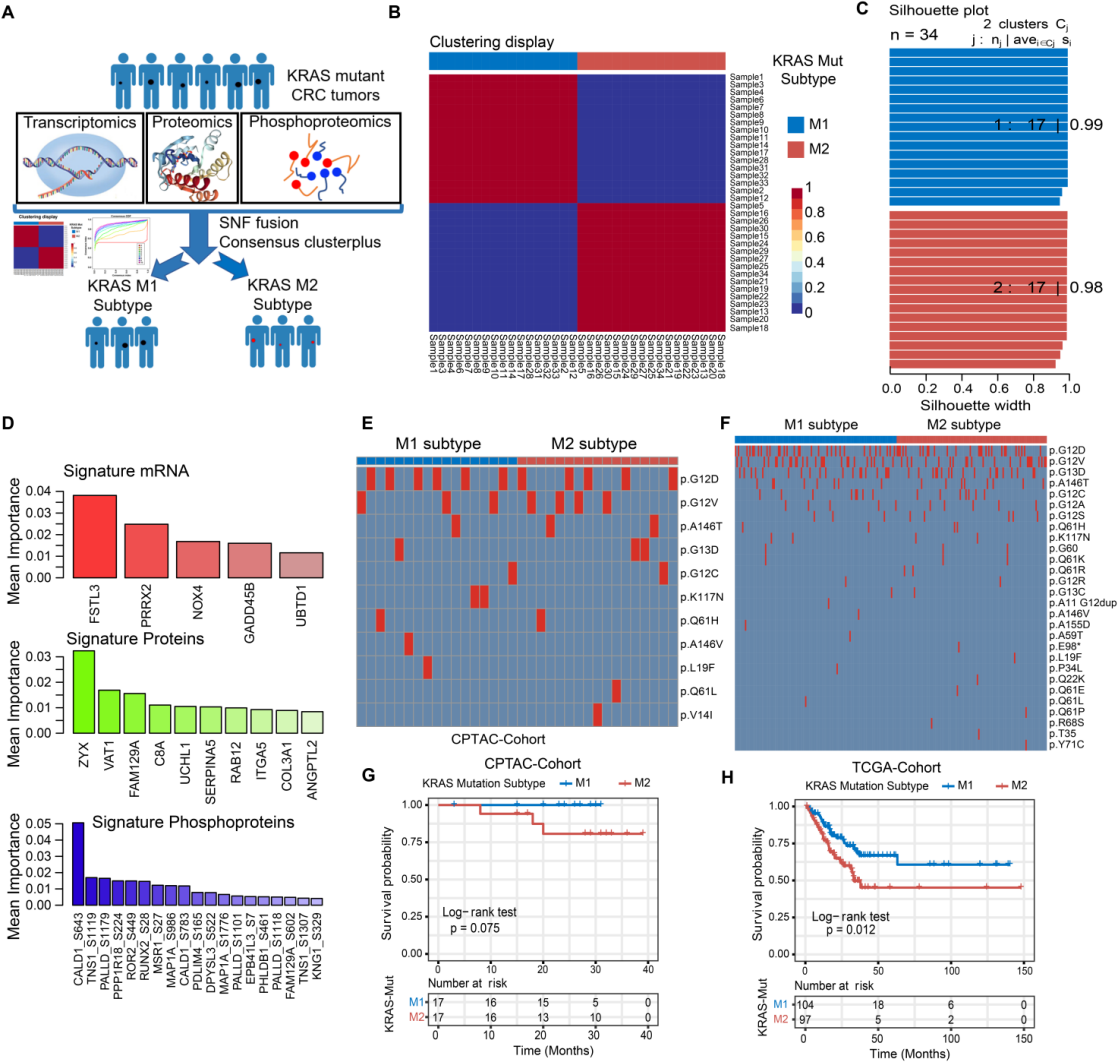
Identifying the genomic and prognostic characterizations of colorectal cancer patients by the molecular subtypes of KRAS**-Mut** subsets. (A) The transcriptomic, proteomic and phosphoproteomic data of KRAS-Mut tumors in CPTAC cohort were integrated by SNF fusion and divide into KM1 subtype and KM2 subtype with unsupervised clustering. (B) Consensus matrix of unsupervised clustering based on the integrative multi-omics data and identify the best cluster number with k=2. (C) The silhouette width of unsupervised clustering based on SNF method in integrated omics data when k = 2. (D) The most discriminative signatures of each datatype (mRNA, protein, and phosphoprotein) selected by random forest. (E) Comparison of the common KRAS mutated residues between KM1 and KM2 subtypes in CPTAC cohort. (F) Comparison of the common KRAS mutated residues between KM1 and KM2 subtypes in TCGA cohort. (G) Kaplan-Meier curves for patients with KM1 and KM2 groups in the CPTAC cohort. (H) Kaplan-Meier curves for patients with KM1 and KM2 groups in the TCGA cohort.

We further integrated publicly available transcriptomic, proteomic and phosphoproteomic data from the CPTAC dataset to investigate the consensus molecular subtype and therapeutic vulnerability underlying the KRAS-Mut tumors (Figure 3A, Table S5). Unsupervised clustering based on three types of omics data with the similarity network fusion (SNF) approach was performed, and KRAS-mutant CRC was classified into two robust subsets (designated KM1 and KM2; Figure 3B, Figure S3A-S3B). Unsupervised clustering was also applied to each separate layer of omics data and showed that the average silhouette width of the subsets from the transcriptome, proteome, or phosphoproteome alone was smaller than that of the subsets from the integrated multi-omics (Figure 3C, Figure S3C), suggesting that integration of the three types of omics data could more accurately classify KRAS-Mut colorectal cancer. To identify the most discriminative molecular signatures between the KM1 and KM2 subtypes, we extracted the most important molecular features to distinguish the subtypes using a random forest learning model. This analysis identified 5 mRNAs, 10 proteins, and 19 phosphoproteins as subtyping signatures at the mRNA, protein, and phosphoprotein levels, respectively (Figure 3D, Figure S3D). To further explore the discrimination ability of the identified features in other independent colorectal cancer datasets, we utilized the identified mRNA signatures in the TCGA, CIT, and GSE87211 cohorts and the protein signatures in the CCRC cohort to perform unsupervised clustering in KRAS-Mut tumors. The expression of the signatures in independent cohorts (TCGA, CIT, GSE87211, and CCRC cohorts) was largely similar to that in the CPTAC cohort (Figure S3E-S3J). We further compared the mutated residues in KRAS (G12D/V/C, G13D, etc.) between the KM1 and KM2 subgroups in CPTAC and TCGA and found no statistically significant differences between the two subtypes (P > 0.05, Fisher's exact test; Figure 3E-3F).

To explore the potential clinical utility of the subtyping signatures, we compared the prognosis between the two molecular subsets. The KM1 subtype in the CPTAC cohort was associated with better prognosis and there are no death outcomes relative to the KM2 subtype, although the survival analysis was not statistically significant considering the small sample size (log-rank test, P = 0.075; Figure 3G). We also compared the prognosis based on KRAS molecular subtype in the independent cohort and determined that the KM1 subtype was significantly associated with better survival in the TCGA, CIT, GSE87211 and CCRC cohorts (Figure 3H, Figure S4A-S4C). Multivariate Cox proportional hazards regression analysis further demonstrated that the KRAS mutation subtype was associated with patient survival outcomes in these KRAS-Mut samples after adjusting for clinicopathologic factors (HR, KM2 vs*.* KM1; TCGA: 1.87 [95% CI, 1.11 to 3.15], P = 0.019; CIT: 2.01 [95% CI, 1.19 to 3.40], P = 0.009; GSE87211: 6.22 [95% CI, 1.40 to 27.59], P = 0.016; CCRC: 3.81 [95% CI, 1.20 to 12.08], P = 0.023; Figure S4D-S4G).

### Tumor genomic variation in KRAS-Mut colorectal cancer

**Figure 4:**
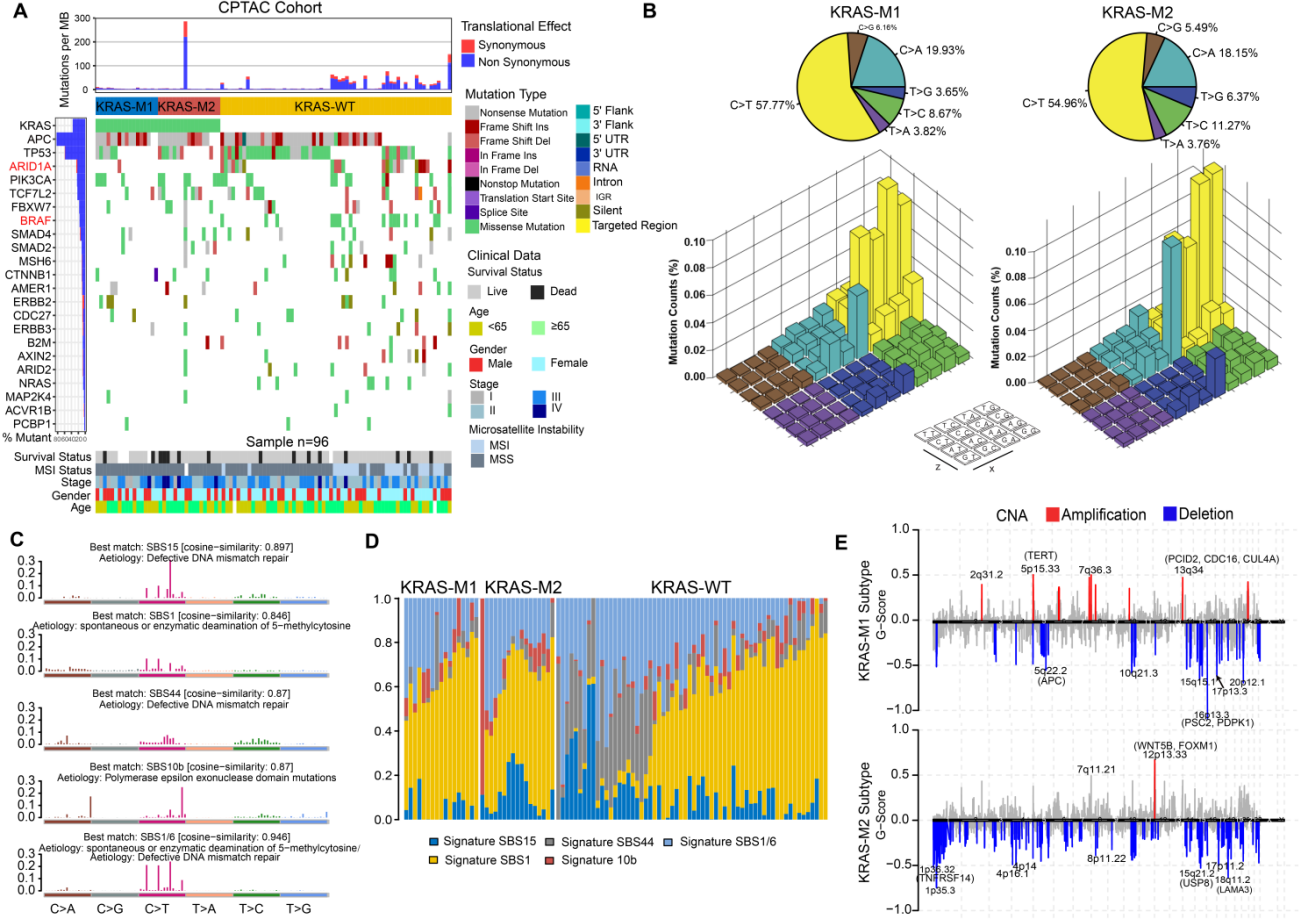
** Tumor genomic landscapes in KRAS-Mut colorectal cancer.** (A) Mutational landscape of SMGs in CPTAC tumors and stratified by KM1, KM2 and WT subgroups. Individual patients were represented in each column. The upper barplot showed mutational load, the right bar plot showed the mutation frequency of each gene in separate groups. Age, stage, gender, MSI status, and survival outcome were shown as patient annotations. (B) Lego plot representation of 96 nucleotide mutation patterns in colorectal cancer samples. Single-nucleotide substitutions were divided into six categories with 16 surrounding flanking bases. The pie chart in upper left showed the proportion of six major categories of nucleotide variation. (C) The mutational activities of corresponding extracted mutational signatures (signature SBS15, SBS44, SBS1/6, SBS1 and SBS10b, named as COSMIC-V3 database). (D) Distribution of mutational counts attributed to corresponding mutational signatures in different KRAS mutation subtypes. (E) Focal peaks with significant somatic copy-number amplification (red) and deletions (blue) (GISTIC2 Q-values < 0.1) are shown. The top ten amplified and deleted cytobands are labeled. Representative genes encoded from these focal peaks are highlighted in approximate positions across the genome.

To gain further insights into the genomic landscape in KRAS-mutated colorectal cancer patients, we parsed the somatic mutation data from WES-seq and SNP array analysis. We performed SMG analysis in the CPTAC and TCGA cohort and compared the gene mutation frequency in the KM1, KM2 and KRAS-WT subsets. The mutational profile in both the TCGA and CPTAC cohorts showed that ARID1A and BRAF had higher mutation rates in the KRAS-WT subgroup than in the KRAS-Mut subgroup (adjusted chi-square test, P < 0.05; Figure 4A, Figure S5A). In the larger-sample-size TCGA cohort, it also showed a higher mutation frequency of APC and PCBP1 in KRAS-Mut samples than in WT tumor samples (adjusted chi-square test, P < 0.05; Figure S5A). Then, we analyzed the single-nucleotide variants (SNVs) in the matrix of 96 possible mutations occurring in a trinucleotide context among the KM1, KM2 and KRAS-WT subtypes in CRC tumors (Figure 4B). The pie chart shows that compared with KRAS-WT tumors, KM1 and KM2 tumors had a slight increase in C>A transitions (Figure 4B, top). The Lego plot shows that the predominant mutations in CRC were C>T transitions at ApCpN trinucleotide sites, whereas the C>A transition at GpCpG sites was specifically highlighted in the KM2 subtype (Figure 4B, bottom), suggesting the specific mutation processes operative in KRAS mutation heterogeneity.

Subsequently, we extracted five mutational signatures from the genomic data (Figure S5B, Figure 4C), including defects in DNA mismatch repair-related signatures (SBS15 and SBS44), spontaneous or enzymatic deamination of 5-methylcytosine (SBS1), and polymerase epsilon exonuclease domain mutation (SBS10b) (Figure 4C). Mutation counts attributed to the SBS44 signature showed a significant increase in KRAS-WT tumors, whereas the SBS15 signature showed a significant decrease in the KM1 subtype (P < 0.05, Figure 4D). We also analyzed the SCNA level in the KM1 and KM2 subgroups and found an obvious elevation in KM1 tumors (Figure S5C). Arm-level SCNA results indicated that the cytobands in chr2, chr5p, chr7q, chr8p, chr12 and chr16 contained the most frequently amplified or deleted regions (Figure S5D). The focal level SCNAs revealed that the cytobands in 2q31.2, 5p15.33, 7q36.3 in KM1 subtype, and 12p13.33 in KM2 subtype contained the markedly amplified focal regions; and cytobands in 16p13.3 in KM1subtype and 1p35.3-36.32, 8p11.22, 18q11.2 in KM2 subtype contained the frequently deleted regions (FDR < 0.05; Figure 4E). The genomic alteration in CNA contributed to the molecular heterogeneity of the KRAS-mutant subtype. Similar genetic variants in SCNA cytobands were also identified in TCGA KRAS-mutated subtype (Figure S5E).

### KRAS mutation patterns characterized by specific clinical features and molecular processes

**Figure 5:**
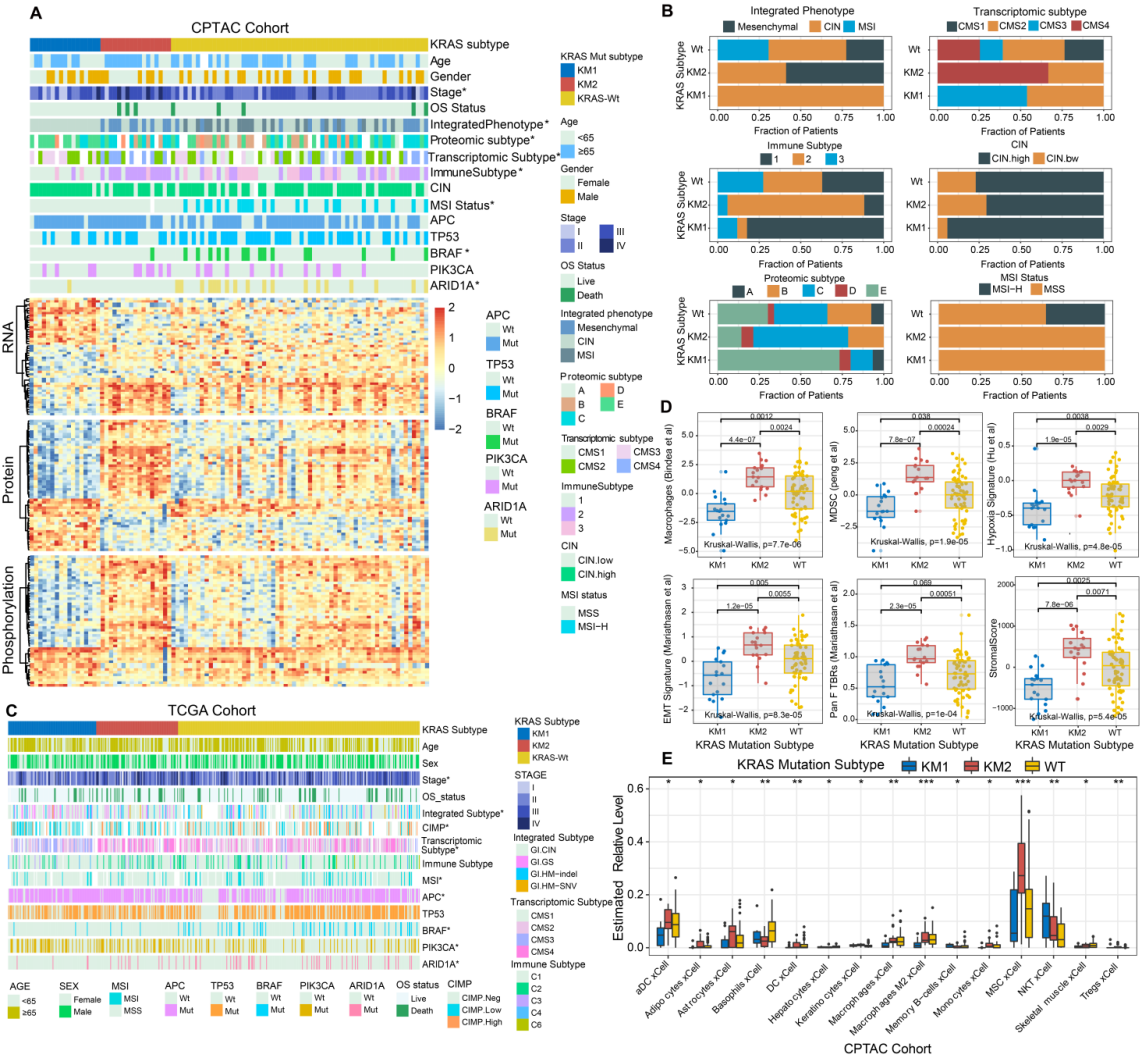
** KRAS-Mut patterns characterized by specific clinical features and molecular processes.** (A) The relationship of clinical characteristics and molecular subtypes among KM1 and KM2 and KRAS-WT in colorectal cancer from CPTAC cohort. The heatmap shows the top 50 differential mRNA transcripts, proteins, and phosphoproteins, for each multi-omics cluster. (B) Comparison of the proportion of CPTAC integrated phenotype, transcriptomic subtype, immune subtype, chromosome instability, proteomic subtype, and MSI status among KM1, KM2 and WT subgroups. (C) The relationship of clinical characteristics and molecular subtypes among KM1 and KM2 and KRAS-WT in colorectal cancer from TCGA cohort. (D) Distribution of curated Immuno-oncology related signatures from Zeng et al study, including Macrophages, MDSC, hypoxia signature, EMT signature, Pan F TBRs, and Stromal score among KRAS-WT, KM1 and KM2 subgroups in CPTAC cohort. (E) Comparison of TME cellular level inferred by xCELL algorithm among KRAS-WT, KM1 and KM2 groups in CPTAC cohort. Within each group, the thick line represents the median value. The bottom and top of the boxes are the 25th and 75th percentiles (interquartile range). The whiskers encompass 1.5 times the interquartile range. The range of P values are labeled above each boxplot with asterisks (*P < 0.05, **P < 0.01, ***P < 0.001).

The relationships of KRAS mutational patterns with clinical characteristics and molecular subtypes were further explored in CRC tumors. The top 50 differential expressed mRNA transcripts, proteins, and phosphoproteins of KRAS-mutant subtypes are shown in a heatmap (Figure 5A). Interestingly, all the MSI-positive tumors were clustered into KRAS-WT subgroup, and resulted in the aggregation of hypermutated phenotype, MSI and immune-related CMS1 transcriptomic subtype, and BRAF and ARID1A mutation. KM2 was associated with advanced tumor stage, the mesenchymal phenotype, the CMS4 transcriptomic subtype, ProS-C proteomic subtype and immune subtype 2 (Figure 5A-5B), which indicated stromal invasion, angiogenesis, and worse prognosis. The KM1 subtype was mainly characterized by early tumor stage, the CIN phenotype, the epithelial and metabolic-related CMS3 transcriptomic subtype, the ProS-E proteomic subtype, and immune subtype 1 (Figure 5A-5B), which suggested epithelial features and better survival outcomes. Similar results were ascertained in the TCGA cohort (Figure 5C).

We further analyzed the tumor biological signatures 39 to explore the representative immune and cancer-related processes within the KRAS-Mut subsets. Among the top six differential molecular signatures, the KM2 subset exhibited the highest enrichment of macrophages, MDSCs, hypoxia signature, EMT signature, Pan-F-TBRs, and stromal score, followed by the KM1 subset and KRAS-WT subset, in the CPTAC cohort (Kruskal-Wallis H test, P < 0.0001, Figure 5D). The ImmuneScore and T cell exhaustion level were also differentially distributed among the KRAS subsets, while there was no statistical significance in the mutation load (Figure S6A). We also performed the identical analyses in the TCGA cohort and obtained the similar results (Figure S6B-S6C). We further adopted the xCell algorithm to digitally portray and dissect the landscape of cellular heterogeneity in tissues of the three KRAS subsets. KM2 was characterized by augmentation in activated dendritic cells (aDCs), adipocytes, astrocytes, M2 macrophages, mesenchymal stem cells (MSCs), etc., and the KM1 subgroup was distinguished by memory B cells and NK cells (Figure 5E).

### Functional annotation of KRAS mutation patterns by proteomic and phosphoproteomic analyses

**Figure 6:**
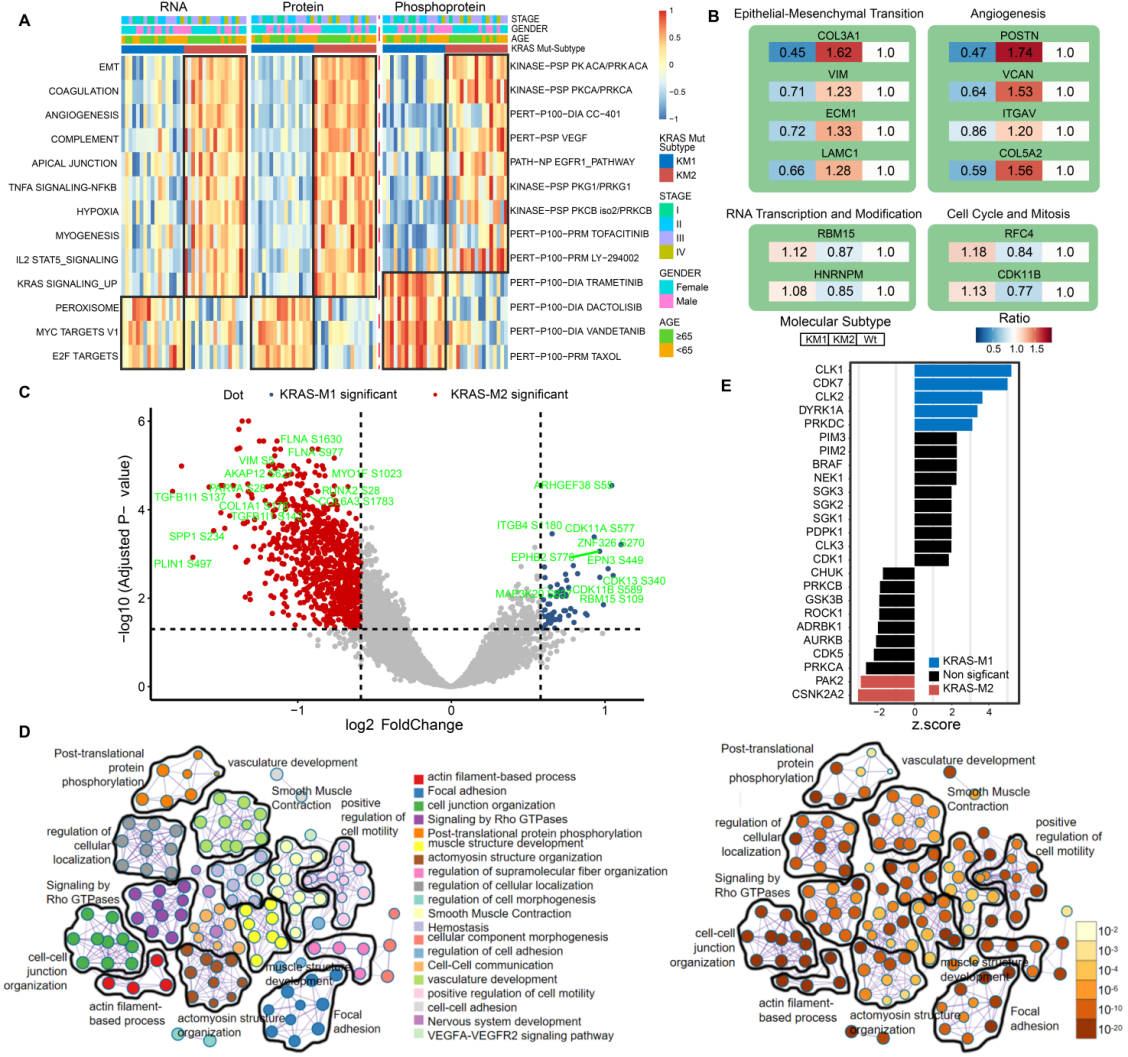
** Functional annotation of KRAS-Mut subtypes by phosphoproteome analysis.** (A) Heatmap shows the representative biological pathways among KM1, KM2 and KRAS-WT subgroups in RNA, protein, phosphoprotein level of CPTAC cohort. (B) The representative differential proteins expression level in KM1 or KM2 versus KRAS-WT, respectively. The color represents the ratio of level in different subgroup. (C) The volcano plot of differential phosphorylation sites in KM1 versus KM2. Blue and red points represented significantly differential phosphoprotein in M1 and M2 subtype, respectively. X-axes showed log2 (fold change) and y-axes showed -log10 (adjusted P value). (D) The expression of phosphorylated protein sites in KM1 and KM2 groups were applied to perform pathway and process enrichment analysis via Metascape. (E) Enriched kinases in KM1 and KM2 subsets using KSEA with a significance of P < 0.05. Blue bars represent enriched in KM1; Red bars enriched in KM2.

Next, to further clarify the biological implications of the KRAS mutation subtype, we performed ssGSEA/post-transcriptional modification (PTM) and Metascape analyses in the KM1 and KM2 tumor subsets at the RNA, protein, and phosphoprotein levels. The pathway enrichment at the RNA and protein levels indicated that KM1 was mainly enriched in the peroxisome, MYC targets, and Wnt β-catenin pathways, whereas KM2 was mainly enriched in the EMT, coagulation, angiogenesis, KRAS signaling-up, and myogenesis processes (Figure 6A). PTM analysis of the phosphoprotein dataset revealed that KM2 was characterized by upregulation of the kinase activity of PKACA, VEGF, PI3K (LY-294002), and JAK (tofacitinib), while KM1 tumors were featured by drug target of MEK (trametinib), PI3K/mTOR (dactolisib), and Taxol. The proteomic data of KRAS-Mut tumors in the CCRC cohort were collected and subjected to ssGSEA, and the results were similar to those in the CPTAC cohort (Figure S7A). Furthermore, the representative proteins of the EMT process (COL3A1, VIM, ECM1, and LAMC1) and angiogenesis pathways (POSTN, VCAN, and ITGAV) were significantly overexpressed in the KM2 subtype (Figure 6B). However, RBM15 and HNRNPM (representative genes related to RNA transcription and modification) and RFC4 and CDK11B (representative genes related to the cell cycle and mitosis) were obviously overexpressed in the KM1 subtype (Figure 6B). We also identified the differentially expressed proteins between KM1 and KM2 tumors and performed protein-protein interaction analysis with the Metascape tool. The resulting network contained the subset of proteins that participate in physical interactions. According to the MCODE algorithm, 18 subclusters of proteins were identified, as shown in Figure S7B; the proteins in each cluster shared the same GO terms and KEGG pathways. Then, we compared the expression of phosphorylation sites in the KM1 and KM2 subgroups in the CPTAC cohort. We found that phosphorylation at the adhesion signaling-related phosphorylation sites FLNA-S1630 and MYO1F-S1023 and the mesenchymal phenotype-related sites VIM-S5, COL1A1-S176, and TFB1I1-S143 was significantly upregulated in the KM2 subgroup. Moreover, the levels of the tyrosine-protein kinase receptor EPHB2-S776 and cell cycle-related proteins CDK11A-S577 and CDK13-S432 were markedly elevated in the KM1 subgroup (Figure 6C, Table S6). Then, we performed pathway and process enrichment analyses based on the differentially phosphorylated sites. We found that many pathways and functions were significantly enriched, such as actin filament-based process, focal adhesion, and signaling by Rho GTPases, and that most of these functions and pathways were mediated by KRAS subtypes (Figure 6D). The proteomic and phosphoproteomic data in the CCRC cohort were retrieved and subjected to Metascape analysis, and the results were similar to those in the CPTAC cohort (Figure S7C-S7F). Further kinase-substrate enrichment analysis (KSEA) 40-42 in the CPTAC and CCRC cohorts revealed that the CDC-like kinases CLK1/2, cyclin-dependent kinases CDK1/7 and extracellular signal-regulated kinases (ERKs) MAPK1/3 were significantly enriched in the KM1 subgroup; however, the apoptosis-related kinases PAK2 and PAK5 and the AKT serine-threonine protein kinase were markedly enriched in the KM2 subgroup (Figure 6E, Figure S7G, Table S7).

### Correlation analysis between molecular features and drug sensitivity reveals subset-specific therapies for KRAS-Mut colorectal cancer

**Figure 7:**
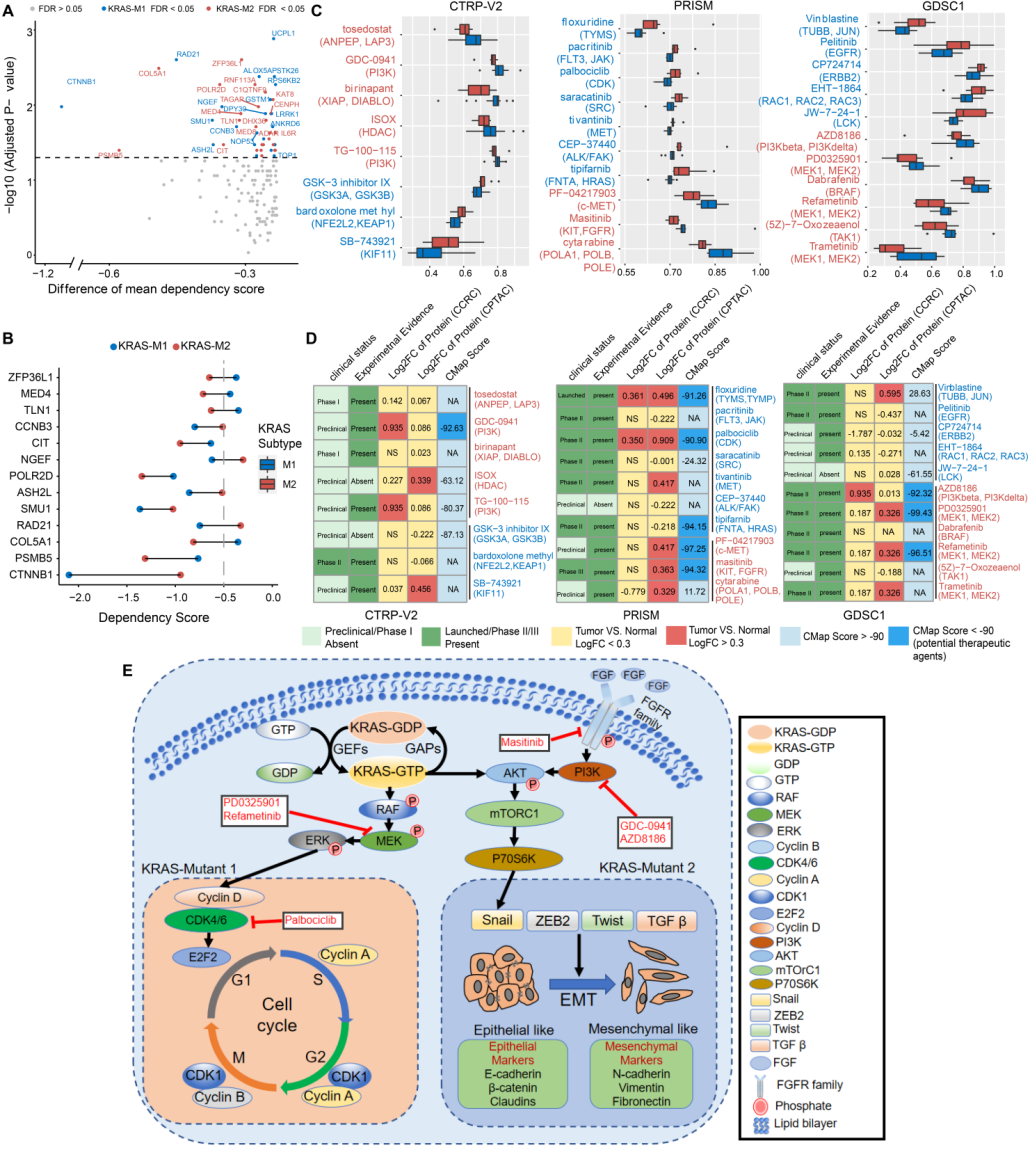
** Correlation between molecular feature and drug sensitivity reveals subsets-specific agents for KRAS-Mut colorectal cancer.** (A) Dot plot showing the differences of gene dependencies between KM1 and KM2 groups. The genes with statistically different dependency scores in each subset are highlighted in blue (M1), and red (M2), respectively. Genes with FDR<0.05 are shown in the dot plot. Right shows the dependency scores for subset specific genes. (B) KRAS subgroup specific genes with dependency scores less than -0.3 were shown. (C) Three drug sensitivity databases (CTRP-V2, PRISM, GDSC1) were used to identify the sensitive of KM1 and KM2 cell line subsets to specific agents. Agents with lower AUC values on the x-axis of boxplots imply greater drug sensitivity. (D) Identification of most promising therapeutic agents for KM1 and KM2 subtype patients according to the evidence from multiple sources. (E) Scheme of signaling cascade and drug targets that are modulated in KRAS-Mut subtypes. The lower left panel displays the cell-cycle and checkpoints pathway that is dysregulated in KM1 patients and the lower right panel displays the epithelial-mesenchymal transition pathway that is dysregulated in KM2 patients.

To explore the potential vulnerability of KRAS-Mut subtypes in CRC, we compared the gene dependencies based on large-scale RNAi screening among the colorectal cancer cell lines in the Cancer Dependency Map (DepMap) dataset. Forty-one KRAS-mutant CRC cell lines were curated and classified into the KM1 or KM2 subset. We identified 31 genes with significantly different dependency scores (mean dependency score difference < -0.25, Wilcoxon rank-sum test, P < 0.05) between the KM1 and KM2 subsets (Figure 7A). Using a mean dependency score of less than -0.3 as a cutoff, we identified 13 genes as subset-specific cancer dependency genes (Figure 7B). Among of them, the KM2 subtype included the PI3K/AKT signaling-related ZFP36L1 and the VEGF signaling-related COL5A1 and TLN1; the KM1 subtype featured the cell cycle/mitosis-dependent CCNB3, RAD21 and the core Wnt signaling molecule CTNNB1.

To further identify the subset-specific therapeutic agents for KRAS-mutant CRC subtypes, we applied three drug response databases (CTRP-v2, PRISM and GDSC1) to investigate the potential therapeutic agents of KM1 and KM2 cell subsets (Figure 7C). Differential drug response analysis between the KM1 and KM2 subgroups was conducted to identify specific compounds with lower estimated AUC values in each group (adjusted P < 0.05). These analyses yielded 8 CTRP-derived compounds (3 for KM1, 5 for KM2) and 10 PRISM-derived compounds (7 for KM1, 3 for KM2) and 11 GDSC-derived compounds (5 for KM1, 6 for KM2) (Figure 7C). Among the common drug targets between the three drug repositories, PI3K inhibitors were identified in both the CTRP and GDC databases for the KM2 subtype (Figure S8A).

Multiple perspective analyses were subsequently conducted to investigate the therapeutic potential of these compounds in CRC. We first used Connectivity Map (CMap) dataset to identify compounds for which the gene expression patterns were opposite the KRAS subtype-specific expression patterns (i.e., gene expression was increased in KM1 or KM2 subtype tumor tissues but decreased after treatment with the compound). Six compounds for the KM2 subtype and three compounds for the KM1 subtype had a CMap score of less than -90 (Figure 7D). We further compared the protein expression patterns of candidate drug targets and retrieved the experimental and clinical evidence to filter the potential drugs (Supplementary Files). Representative results are presented in Figure 7D and Table S8. In general, PI3K/AKTi, MEKi and FGFRi, which had robust *in vitro* and *in silico* evidence, were considered to hold the most promising therapeutic potential in patients with KM2 CRC. Moreover, floxuridine and CDKi hold the promising therapeutic potential in patients with KM1 CRC. We also explored the potential gene regulators that highly correlated with sensitivity to MEKi, ERKi or AKTi in each KRAS subtype, which may be potential indicators for the response to such inhibitors (Figure S8B). Finally, we generated a schematic summarizing the signaling pathways in the KRAS-Mut subtype to outline our findings in KRAS-mutant CRC (Figure 7E).

## Discussion

KRAS is frequently mutated in CRC and is involved in the occurrence, progression, immune evasion, and treatment resistance of CRC. Due to the broad heterogeneity of KRAS mutations, this group of CRC patients requires more precise and personalized treatment. In this study, genomic, transcriptomic, proteomic and phosphoproteomic analyses were leveraged to explore the clinicopathologic features and molecular networks in KRAS-Mut CRC tumors. Single-cell RNA-seq was also employed to delineate the TME cellular landscape of KRAS mutant CRC tumors. Our integrative analysis intrinsically separated KRAS-mutant tumors into two subtypes, KM1 and KM2, which are characterized by different genomic variations, survival outcomes and biological phenotypes. We further utilized a large-scale drug screening system based on KRAS-mutant CRC cell lines and identified potential therapeutic agents for the specific KRAS-Mut subtypes. Our study confirmed the value of proteogenomic integration in uncovering novel cancer biological implications of oncogenic driver KRAS mutations and further demonstrated the utility of proteogenomics in guiding therapeutic regimens.

Previous studies reported that cytotoxic T cells, neutrophils and the interferon gamma pathway were suppressed in KRAS-Mut tumors 7, 8. Our preliminary analyses also found similar results and further indicated that Tregs, MDSCs and epithelial cells were increased in KRAS-Mut tumors. The mutational profiles at the single-cell level were more precise in evaluating the cellular landscape of KRAS-mutated CRC. However, the current technology bottleneck of simultaneous sequencing of genome and transcriptome in individual cells limits the accurate exploration of cellular heterogeneity. Therefore, this study utilized a compromise approach in which the tissues were divided into two parts: half of the tissues were used for single-cell isolation, and the other half were prepared for DNA extraction and mutation testing. Finally, we utilized the single-cell RNA-seq data to compare the difference of TME cell-infiltrating landscape based on bulk WES-identified KRAS-WT and KRAS-Mut colorectal tumors. The scRNA-seq analyses revealed alterations in specific cell subpopulations (including SPP1^+^ macrophages, myofibroblasts and IgG^+^ plasma cells) related to KRAS mutation and suggested the roles of genetic alterations in tumor cells that determine the formation of these unique tumor microenvironments in CRC. Giopanou et al*.* found that lung macrophages and epithelium-secreted SPP1 drove tumor-associated inflammation, while SPP1 promoted early tumorigenesis by promoting the survival of KRAS-mutated cells, especially in KRAS-G12D-driven tumors 43. Stromal myofibroblasts were reported to promote the development of pancreatic cancer via collaboration with the epithelial compartment harboring oncogenic Kras mutations 44. The characterization of cellular profiles in the TME of CRC implies the immunomodulatory effect of KRAS mutation, particularly via promotion of inflammation and evasion of the immune response, ultimately leading to tumor progression, invasion, and progression.

Considering the inferior clinical outcomes of KRAS-mutant CRC tumors, we performed an integrative clustering analysis to better stratify patients for therapeutic interventions and provide a more precise assessment of phenotypic heterogeneity. The KM2 subtype was associated with the mesenchymal phenotype-related CMS4 subtype and enriched in EMT, TGF-β and angiogenesis pathways, which indicated stromal invasion and worse prognosis. Studies have revealed that EMT-related molecules promote KRAS-driven tumor development and suppress sensitization to MEK inhibitors 44, 45. Moreover, Adachi et al*.* confirmed that EMT is a cause of both intrinsic and acquired resistance to KRAS G12C inhibitors in KRAS G12C-mutant non-small-cell lung cancer 46, suggesting that the EMT process plays a critical role in KRAS mutation-driven tumorigenesis and therapeutic resistance. The KM1 subtype was mainly characterized by the metabolism-related CMS3 subtype and exhibited high activation of cell cycle, MYC and RNA transcription pathways, suggesting cyclin-dependent features and better survival outcomes. Kazi et al. utilized global phosphoproteomic screening and found that CDK was a therapeutic target and that CDK inhibitors can be utilized into the treatment of KRAS-Mut tumors 47. Our KRAS molecular subtyping was also validated in independent KRAS-Mut datasets of CRCC, TCGA and CIT. These results provide instructive molecular insights and indicate the prognostic utility of the identified subtype signature in KRAS-Mut colorectal cancer.

Interestingly, the two KRAS mutational molecular subtypes exhibited few differences in driver gene mutations. However, the copy number variations in cytobands of chr2p, chr5p, chr12p, etc. indicated significant alteration of amplification or deletion between the two KRAS subtypes. Cytoband amplification in the chr5p15.33 region contained the TERT gene, which is related to the cell cycle and regulation of Wnt-beta catenin signaling. Chr5q22.2 contains the tumor suppressor gene APC, whose deletion or inactivation leads to activation of the Wnt signaling pathway and loss of a tumor-suppressive function. Overexpression of WNT5B and FOXM1, located on chr12p13.33, has been reported to be associated with cancer cell migration and proliferation 48, 49. Deletion of chr18q11.2 is predictive of survival in patients with metastatic colorectal cancer treated with VEGFR inhibitors 50.

Despite the breakthroughs in allele-specific inhibitors targeting KRAS-G12C mutant cancer 9, direct targeting of KRAS remains extremely difficult 5, 51. Moreover, KRAS-G12D and KRAS-G12V remain the most common mutations in CRC and are found in the largest patient populations 4. Therefore, we utilized three drug sensitivity prediction resources and further filtering with molecular perturbation datasets and clinical/experimental evidence to investigate potential treatment strategies for KRAS mutant CRC. Previous whole-genome CRISPR screening demonstrated the fibroblast growth factor receptor 1 (FGFR1) as the top-ranked target that promoting the survival of mesenchymal phenotype-related cancer cells 52. Manchado et al. indicated that genetic or pharmacological inhibition of FGFR1 in combination with MEKi enhances tumor cell death *in vitro* and *in vivo* 53. Moreover, the combination of floxuridine and CDKi (palbociclib) has promising therapeutic potential in patients with KM1 CRC. Oncogenic KRAS mutations have been demonstrated to trigger a diverse range of phosphorylation cascades, including canonical MAPK, PI3K/AKT and RALGDS/RAL pathways, and inhibitors targeting these molecules have been utilized and combined in clinical practice to suppress these signaling cascades in colorectal cancer 54, 55. Recent studies reported that CDK inhibitors served as the vulnerability and therapeutic targets to KRAS-Mut cancers 47, 56, 57. Testing of colorectal patient-derived xenograft (PDX) models further showed that combination of MEKi and palbociclib (CDKi) was well tolerated and highly efficacious in KRAS-Mut models 56. Recent studies have revealed that phosphorylation of SHP2 (PTPN11) contributes to its interaction with growth factor receptor-bound protein 2 (GRB2) and that it acts as a scaffold protein to recruit the GRB2-SOS complex, thereby promoting RAS nucleotide exchange and cell proliferation 58. In our drug prediction system, inhibitors targeting SHP2 or SOS1 were not statistically significant in KRAS mutational subtype (KM1 vs. KM2), possibly because the compound screening was based on KRAS-Mut samples and the discriminatory effect between KRAS-Mut and WT was weakened.

The clinical relevance of the identified molecular signatures for subtyping was largely validated at the mRNA level but not at the protein/phosphoprotein level because of the limited clinical proteomics data for KRAS-Mut patients. Therefore, further validation of the molecular subtype classification in a large-scale prospective clinical cohort of patients with CRC is required. Furthermore, potential drug combination intervention strategies in specific KRAS-mutant CRC subsets are expected to be further explored in xenograft models and clinical trials. The representativeness of the signatures and drug sensitivities for CRC *in situ* remains to be further validated in large-scale primary samples.

In summary, our integrated proteogenomic characterization revealed new molecular subtypes and therapeutic opportunities for targeting signaling proteins, phosphosites, and genomic alterations in KRAS-mutant colorectal cancer. Although validation of these therapeutic hypotheses is beyond the scope of our current study, our characterization of molecular phenotypes and mutation profiles may enable substantial advances in revealing the molecular heterogeneity of KRAS-Mut tumors. We hope that both the specific observations and hypotheses delineated in this manuscript may lead to the development of more promising KRAS-targeted approaches.

## Supplementary Material

Supplementary figures and table legends.

Supplementary tables.

## Abbreviations

CRC: colorectal cancer
TME: tumor microenvironment
EMT: epithelial-mesenchymal transition
DEGs: differential expressed genes
SCNA: somatic copy number alteration
SMG: significantly mutated genes
TCGA: The Cancer Genome Atlas
CCLE: Cancer Cell Line Encyclopedia
CCRC: Chinese Colorectal Cancer
GSVA: gene set variation analysis
AUC: area under the dose-response curve
TIL: tumor-infiltrating lymphocyte
MDSC: Myeloid-derived suppressor cell
Pan F TBRs: pan-fibroblast TGF-β response signature
PTM: post-transcriptional modification
KSEA: Kinase-substrate enrichment analysis
