# Supplementary material for: Integrated multi-omics characterization of KRAS mutant colorectal cancer
Source: Theranostics. 2022 Jul 4;12(11):5138–54. doi: 10.7150/thno.73089 (PMC9274732; doi:10.7150/thno.73089)
Supplement: Supplementary file 1 — Supplementary figures and table legends. [file thnov12p5138s1.pdf]

Supplementary Information

Collection and curation of proteome and phosphoproteome datasets.

The proteome and phosphoproteome were mainly derived from CPTAC and CCRC cohorts. Briefly, the global proteomics of CPTAC cohort were subjected to TMT10-labeld LC-MS/MS analysis and resulted in a total of 8067 common proteins. The relative protein abundances were log2 transformed and zero-centered for each gene to obtain final, relative abundance values. The TMT-10 phosphoproteome data were processed by the Ascore algorithm[1] for phosphorylation site localization, and the top-scoring sequences were reported. The hybrid spectral library of CCRC cohort proteome or CCRC phosphoproteome was generated by DDA and DIA strategies. Quantity was determined on MS/MS level using area of XIC peaks with enabled cross run normalization and presented a total of 8450 quantified proteins and 47786 phosphosites in CCRC dataset. Detection of clinicopathologic features and MSI status was performed as described in the original reports describing the cohorts.

Analysis of mutational signatures and SCNA

The somatic mutation and copy number alteration (SCNA) segments data of CPTAC and TCGA-COAD/READ were downed and curated for the genomic analysis. “ExtractSignatures” function based on Bayesian variant nonnegative matrix factorization, factorized the mutation portrait matrix into two nonnegative matrices ‘signatures’ and ‘contributions’, where ‘signatures’ represent mutational processes and ‘contributions’ represent the corresponding mutational activities. The extracted mutational portrait of CRC was compared and annotated

by cosine similarity analysis against the Catalogue of Somatic Mutations in Cancer (COSMIC V3). The 96 types single nucleotide variants on CRC genomic landscape were profiled by Lego plot. Moreover, we set up a threshold of 0.4 (*-ta* and *-td* parameters of GISTIC2) in filtering the amplified or deleted regions based on the distribution of germline copy number variants. GISTIC2 generated arm level and focal level SCNAs for the cohort with G-Score and FDR-Q value indicating the significance and strength of the identified SCNAs.

Single cell analyses of CRC cellular landscape

The single cell RNA-seq and metadata were curated form the Samsung Medical Center (SMC cohort)[2] and Katholieke Universiteit Leuven (KUL cohort)[3]. Briefly, gene expression matrix from the CellRanger pipelines was filtered and normalized using the Seurat R package[4] within the following criteria: >1,000 unique molecular identifier (UMI) counts; >200 genes and <6,000 genes; and <20% of mitochondrial gene expression in UMI counts. We further utilized the reciprocal PCA (RPCA) implemented in Seurat (v4.0) to integrate and align the tumor cells from the two scRNA datasets. After the integration, to perform dimension reduction, we first scaled the data by shifting and scaling the expression of each gene so that the mean expression across cells was 0 and the variance across cells was 1. Afterwards we ran PCA analysis and clustered and visualized the aligned dataset using UMAP projection. The major cell types in the datasets have been annotated by comparing the canonical marker genes and the differentially expressed genes (DEGs) for each cluster.

Data Imputation

Missing values of preprocessed RNA-seq, protein and phosphoproteome data were imputed by KNN method using R package “impute”. For protein data, genes presented in at least 70% samples were reserved with the imputation parameters: k = 5, rowmax = 0.3, colmax = 0.4. For phosphoproteome data, phosphosites presented in at least 50% samples were reserved according to previous report[5] with the imputation parameters: k = 5, rowmax = 0.3, colmax = 0.4. For RNA-seq data, genes presented in at least 80% samples were used for data analysis as previously described[6]. The imputed data described above was z-scored for each sample

Consensus Molecular Clustering

We adopted the mRNA, imputed proteomic data and imputed phosphoprotein data to a similarity matrix using R package “CancerSubtypes”[6, 7]by default parameters. The similarity matrix was used as the input of unsupervised clustering performed by R package “ConsensusClusterPlus”[8] with the parameters: maxK = 10, reps = 500, clusterAlg = “spectralAlg.” The number of clusters was demonstrated by the stable shape and maximum area of the consensus cumulative distribution function (CDF) curve with the clearest consensus matrix and the rapid decrease of average silhouette from k = 2 to 4. NMI values for three data types were calculated by the function “rankFeaturesByNMI” in the R package “SNFtool” using default parameters. Besides, each filtered data matrix was used for consensus clustering respectively by R package “ConsensusClusterPlus” with the parameters: maxK = 10, reps = 500, clusterAlg = “km”, distance = “euclidean”.

Variable selection analysis and subset prediction

Variable selection analysis was used for KRAS mutant subset signature selection performed by the R package “VSURF” with random forest algorithm. The number of trees was set to 10,000 as previously described. mRNA data of selected signature from interpretation step was used to build a prediction model for KRAS-WT sub-groups performed by the function “predict” in the R package “VSURF” with the parameters: type = “class”, step = “interp.” The subsets of KRAS mutant (nonsynosmous mutation) subtype in TCGA and CCLE datasets were acquired by unsupervised clustering based on the mRNAs signature using the imputed RNA-seq data.

ssGSEA and PTM-SEA analysis

We utilized gene-centric single sample Gene Set Enrichment Analysis (ssGSEA) of gene expression data (e.g. mRNAs, proteins) and site-centric PTM Signature Enrichment Analysis (PTM-SEA) of phosphoproteomics data sets with the PTM signatures database (PTMsigDB)[9] to investigate the variation in biological processes among different KRAS mutant subtype. The well-defined biological signatures were derived from the Hallmarker gene set (download from MSigDB database v7.1), Zeng et al. curated Immuno-Oncology gene sets[10]. The Student’s t test was used for P value calculation and the ratio of mean was used for fold-change compared to other two subsets. GO annotation for KRAS mutant versus WT subtype-related genes was performed in the R package ‘clusterProfiler’ with the cutoff value of FDR < 0.001.

Metascape and KSEA analysis

Metascape is a web-based portal designed to provide a comprehensive pathway annotation and analysis resource to deconstruct the molecular mechanisms underlying a biological system within OMICs database[11]. Here, we utilized the differential gene expression list (RNA, protein, phosphoprotein) as input to Metascape portal and followed by the analysis guidelines with the cutoff of $P <0.05$ to investigate the variation in biological processes among different KRAS mutant subtype. Kinase-substrate enrichment analysis (KSEA) were performed by KESA App website (https://casecpb.shinyapps.io/ksea/) using phosphosite data according to its manual with the cutoff of $P < 0.05$ and substrate count more than 1.

Colorectal cancer cell line and drug sensitivity analyses

The CTRP (v.2.0, released October 2015), PRISM Repurposing dataset (19Q4, released December 2019) and GDSC1 (Genomics of Drug Sensitivity in Cancer Project, Release 8.1, Oct 2019) for cancer cell line drug sensitivity analyses were achieved from the dependency map (DepMap) portal (https://depmap.org/portal/). The three datasets utilized the area under the dose–response curve (AUC) values as a measure of drug sensitivity, and lower AUC values indicated increased sensitivity to drugs treatment. Before analysis, we firstly removed the agents with more than 20% of missing data, and the remaining drug missing data were also imputed by ‘impute’ R package. For the filtration of the potential drug, fold-change differences of the protein expression levels of candidates’ drug targets between tumor and normal tissue were calculated in CCRC and CPTAC cohort. A higher fold change value indicated a greater potential of candidate agent for CRC treatment. Thirdly, a comprehensive

literature search was performed in PubMed (https://www.ncbi.nlm.nih.gov/pubmed/) and ClinicalTrials website (https://clinicaltrials.gov/) to find out the experimental and clinical evidence of candidate compounds in treating gastrointestinal tumors.

Reference:

1. Beausoleil SA, Villen J, Gerber SA, Rush J, Gygi SP. A probability-based approach for high-throughput protein phosphorylation analysis and site localization. Nat Biotechnol. 2006;24:1285-1292.
2. Lee HO, Hong Y, Etlioglu HE, Cho YB, Pomella V, Van den Bosch B, Vanhecke J, Verbandt S, Hong H, Min JW, et al. Lineage-dependent gene expression programs influence the immune landscape of colorectal cancer. Nat Genet. 2020;52:594-603.
3. Qian J, Olbrecht S, Boeckx B, Vos H, Laoui D, Etlioglu E, Wauters E, Pomella V, Verbandt S, Busschaert P, et al. A pan-cancer blueprint of the heterogeneous tumor microenvironment revealed by single-cell profiling. Cell Res. 2020;30:745-762.
4. Hao Y, Hao S, Andersen-Nissen E, Mauck WM, 3rd, Zheng S, Butler A, Lee MJ, Wilk AJ, Darby C, Zager M, et al. Integrated analysis of multimodal single-cell data. Cell. 2021;184:3573-3587 e3529.
5. Mertins P, Mani DR, Ruggles KV, Gillette MA, Clauser KR, Wang P, Wang X, Qiao JW, Cao S, Petralia F, et al. Proteogenomics connects somatic mutations to signalling in breast cancer. Nature. 2016;534:55-62.
6. Liu K, Guo J, Liu K, Fan P, Zeng Y, Xu C, Zhong J, Li Q, Zhou Y. Integrative analysis reveals distinct subtypes with therapeutic implications in KRAS-mutant lung adenocarcinoma. EBioMedicine. 2018;36:196-208.
7. Wang B, Mezlini AM, Demir F, Fiume M, Tu Z, Brudno M, Haibe-Kains B, Goldenberg A. Similarity network fusion for aggregating data types on a genomic scale. Nat Methods. 2014;11:333-337.
8. Wilkerson MD, Hayes DN. ConsensusClusterPlus: a class discovery tool with confidence assessments and item tracking. Bioinformatics. 2010;26:1572-1573.
9. Krug K, Mertins P, Zhang B, Hornbeck P, Raju R, Ahmad R, Szucs M, Mundt F, Forestier D, Jane-Valbuena J, et al. A Curated Resource for Phosphosite-specific Signature Analysis. Mol Cell Proteomics. 2019;18:576-593.
10. Zeng D, Ye Z, Shen R, Yu G, Wu J, Xiong Y, Zhou R, Qiu W, Huang N, Sun L, et al. IOBR: Multi-Omics Immuno-Oncology Biological Research to Decode Tumor Microenvironment and Signatures. Front Immunol. 2021;12:687975.
11. Zhou Y, Zhou B, Pache L, Chang M, Khodabakhshi AH, Tanaseichuk O, Benner C, Chanda SK. Metascape provides a biologist-oriented resource for the analysis of systems-level datasets. Nat Commun. 2019;10:1523.

Supplementary Figures

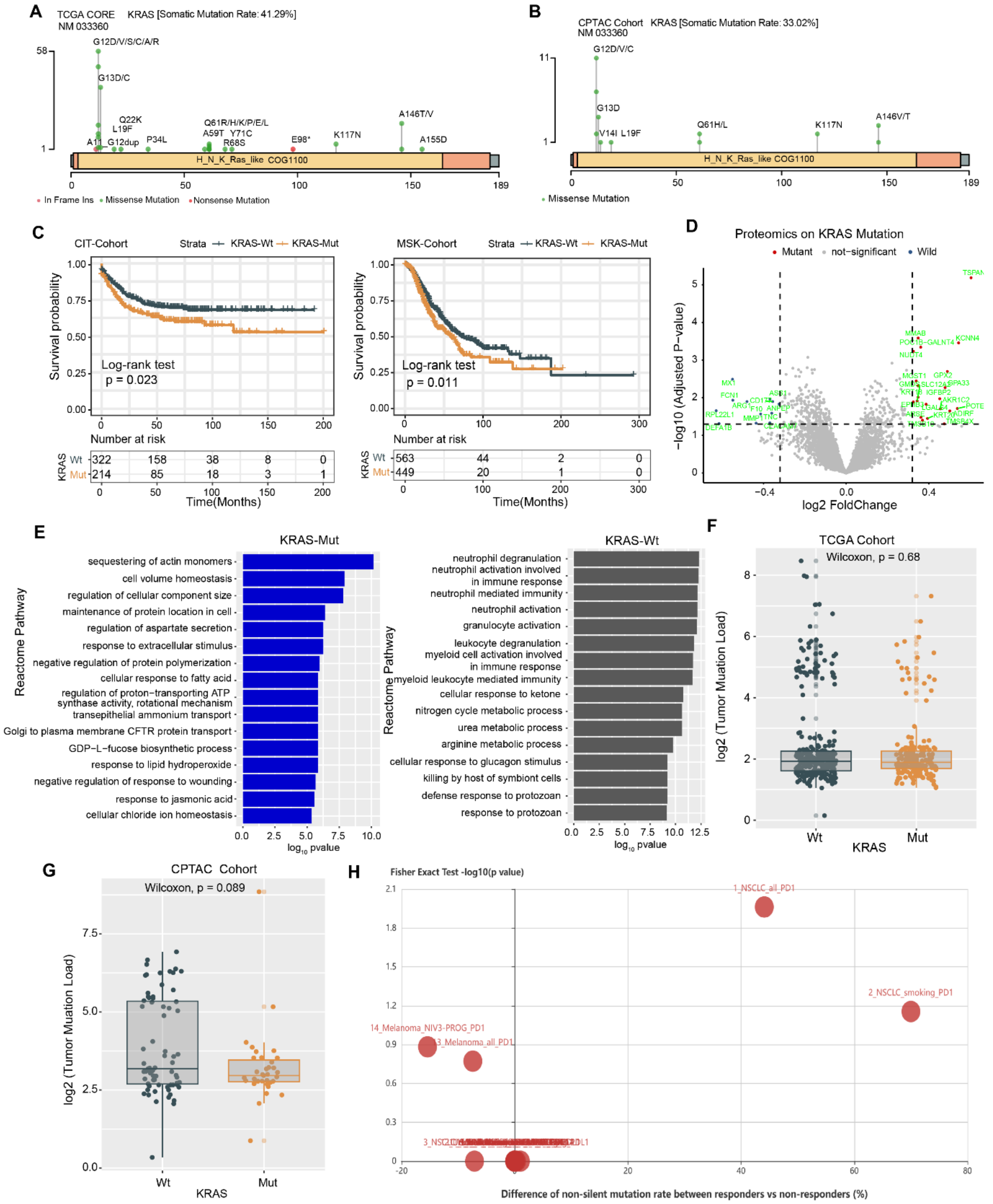

Figure S1. Molecular characteristics of KRAS mutation in CRC

(A-B) KRAS mutational residues in TCGA-COAD/READ and CPTAC cohort were shown by lollipop plot. (C) Kaplan-Meier curves for patients with KRAS-Mut and KRAS-WT groups in the CIT (GSE39582) and MSK cohorts. (D) The volcano plot: green and gray

points represented differentially expressed proteins and no statistically significant difference genes, respectively, in the KRAS-Mut versus KRAS-WT. X-axes showed log2 (fold change) and y-axes showed -log10 (P value). (E) Functional annotation for KRAS-Mut phenotype-related genes using GO enrichment analysis. The color depth of the barplots represented the statistical significance of enriched pathways. (F-G) Comparison of tumor mutation load in KRAS-Mut versus KRAS-WT in TCGA and CPTAC cohorts. (H) The association of KRAS mutation with response to ICB therapy in various tumors types.

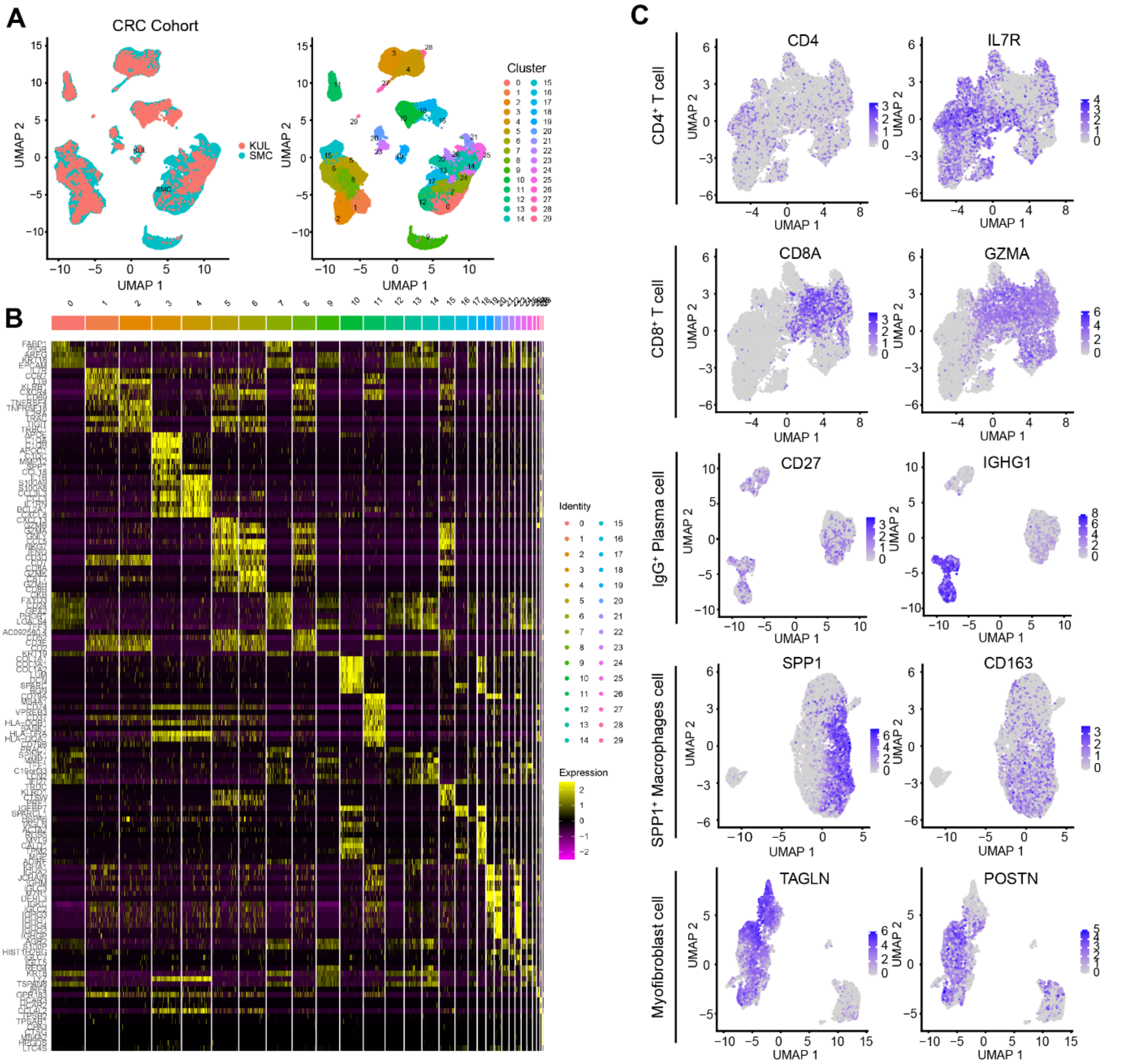

Figure S2. Single-cell transcriptomes profile and representative marker of each cluster with KRAS-Mut colorectal cancer.

(A) UMAP clustering of the aggregated 55539 colorectal cancer cells obtained from 23 SMC and 6 KUL samples colored by the two dataset (left) and 29 clusters (right). (B) Heatmap of the top five significant DEGs for each cluster in CRC tumors. Significant P-values were obtained from the two-sided Student's t-test. (C) The color-coded expression of key markers of CD4+ T cells, CD8+ T cells, IgG+ plasma cells, SPP1+ macrophages cells and Myofibroblast cell in CRC.

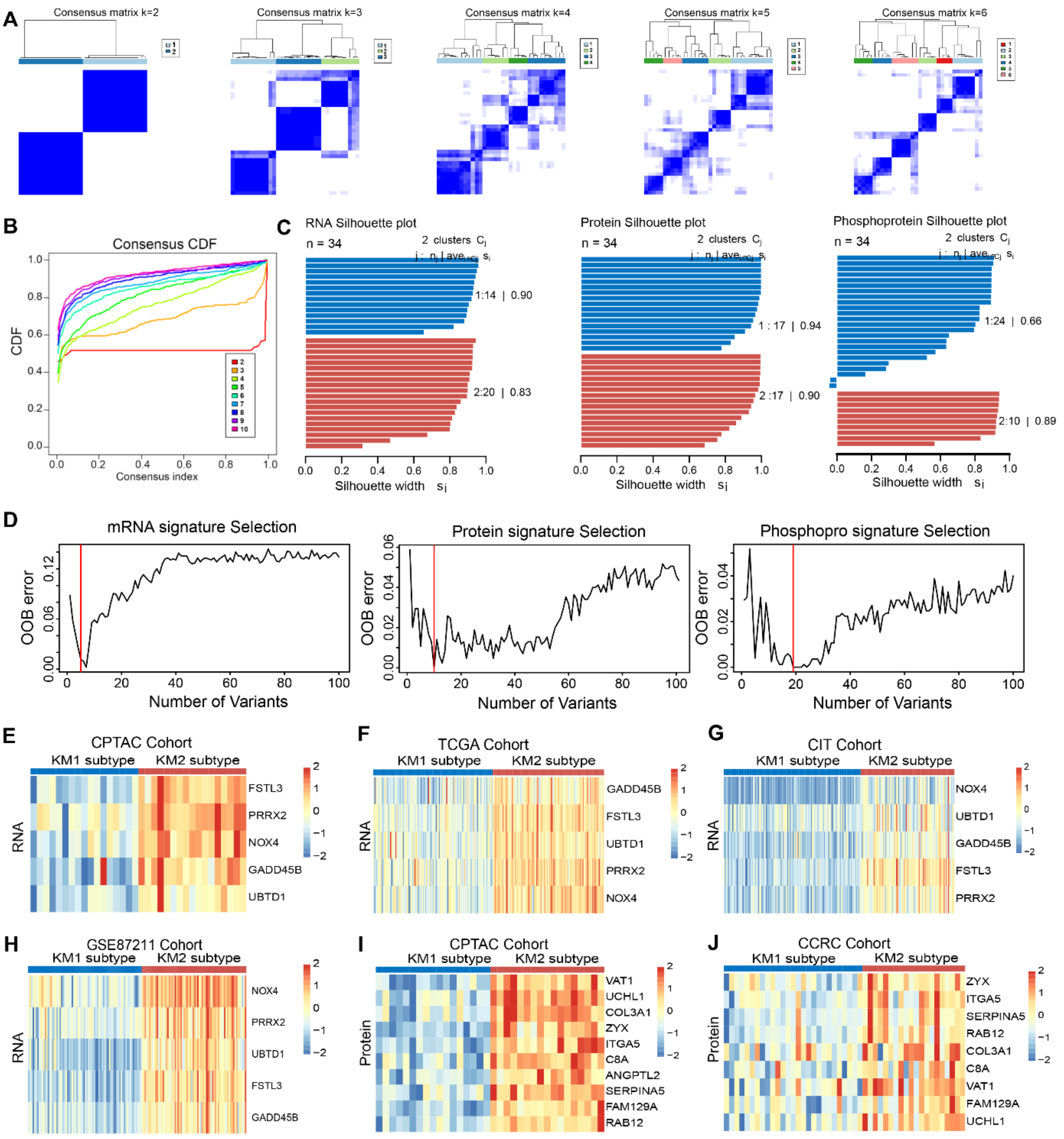

Figure S3. Proteomic and phosphoproteomic analysis enables multi-omics-based integrative subtyping of KRAS-Mut CRC tumors.

(A) Heatmap representation of unsupervised clustering of the multi-omic data in KRAS-Mut tumors of CPTAC cohort with cluster numbers from 2 to 6. (B) The consensus CDF of unsupervised clustering based on multi-omic data. (C) The silhouette width of unsupervised clustering based on SNF method in RNA, Protein and Phosphoprotein, respectively. (D) The

OOB (out of bag) error rate during the signature selection for mRNA, protein, and phosphoprotein using random forest. (E-H) Heatmap of signature mRNAs in KRAS-Mut tumors of CPTAC, TCGA and CIT cohort. (I-J) Heatmap of signature Proteins in KRAS-Mut types of CPTAC and CCRC cohort.

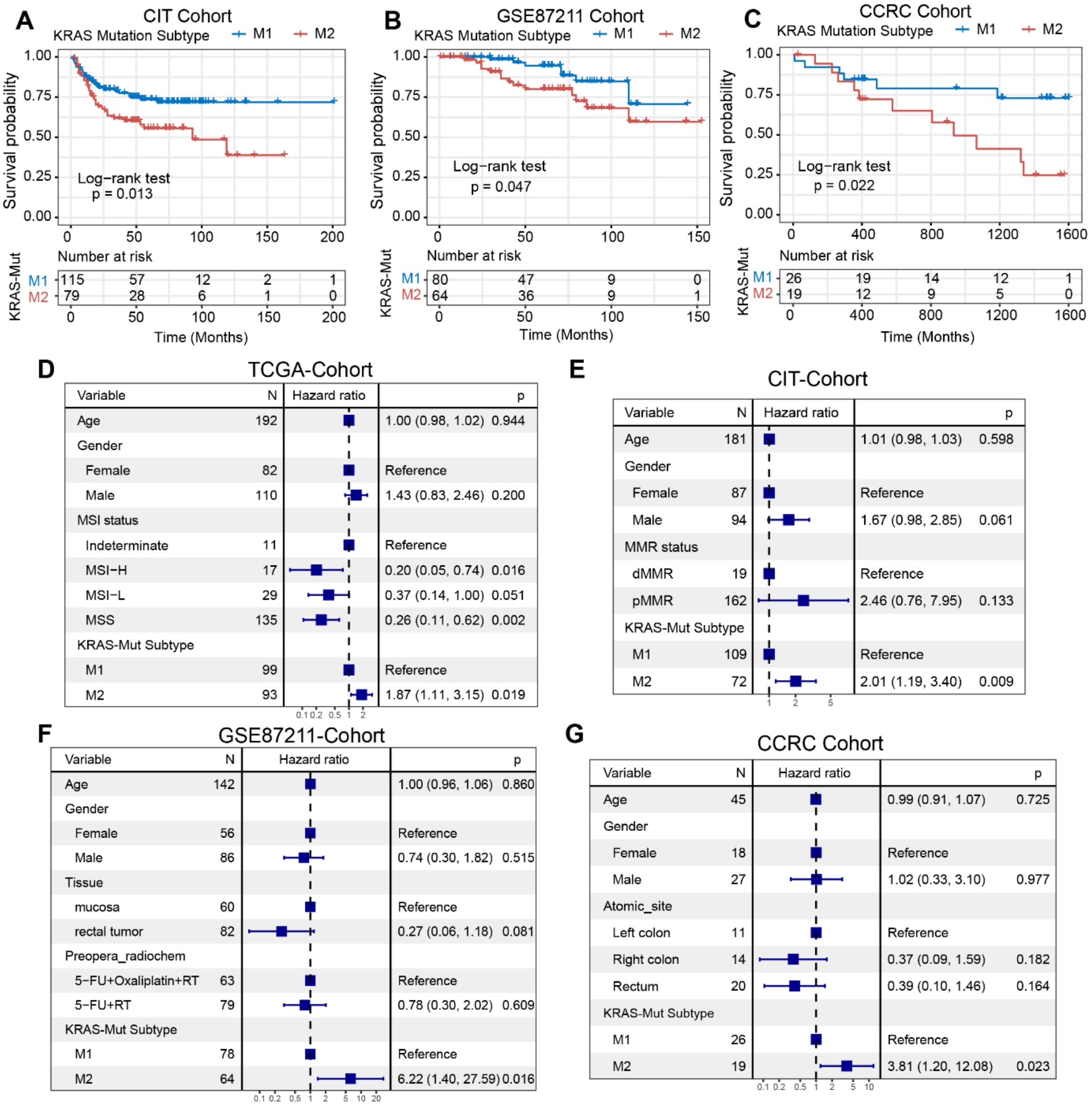

Figure S4. Validation of the prognostic significance of KRAS molecular subtype in independent KRAS-Mut CRC cohorts.

(A-C) Prognosis analysis of the KM1 and KM2 molecular subtype in CIT/GSE39582 cohort (A), GSE87211 cohort (B), and CCRC cohort (C). (D-G) Subgroup analysis estimating clinical prognostic value between KRAS-Mut subtype in TCGA cohort (D), CIT cohort (E), GSE87211 cohort (F), and CCRC cohort (G) by multivariable Cox regression. The length of the horizontal line represented the 95% confidence interval for each group. The vertical dotted line represented the hazard ratio (HR) of all patients.

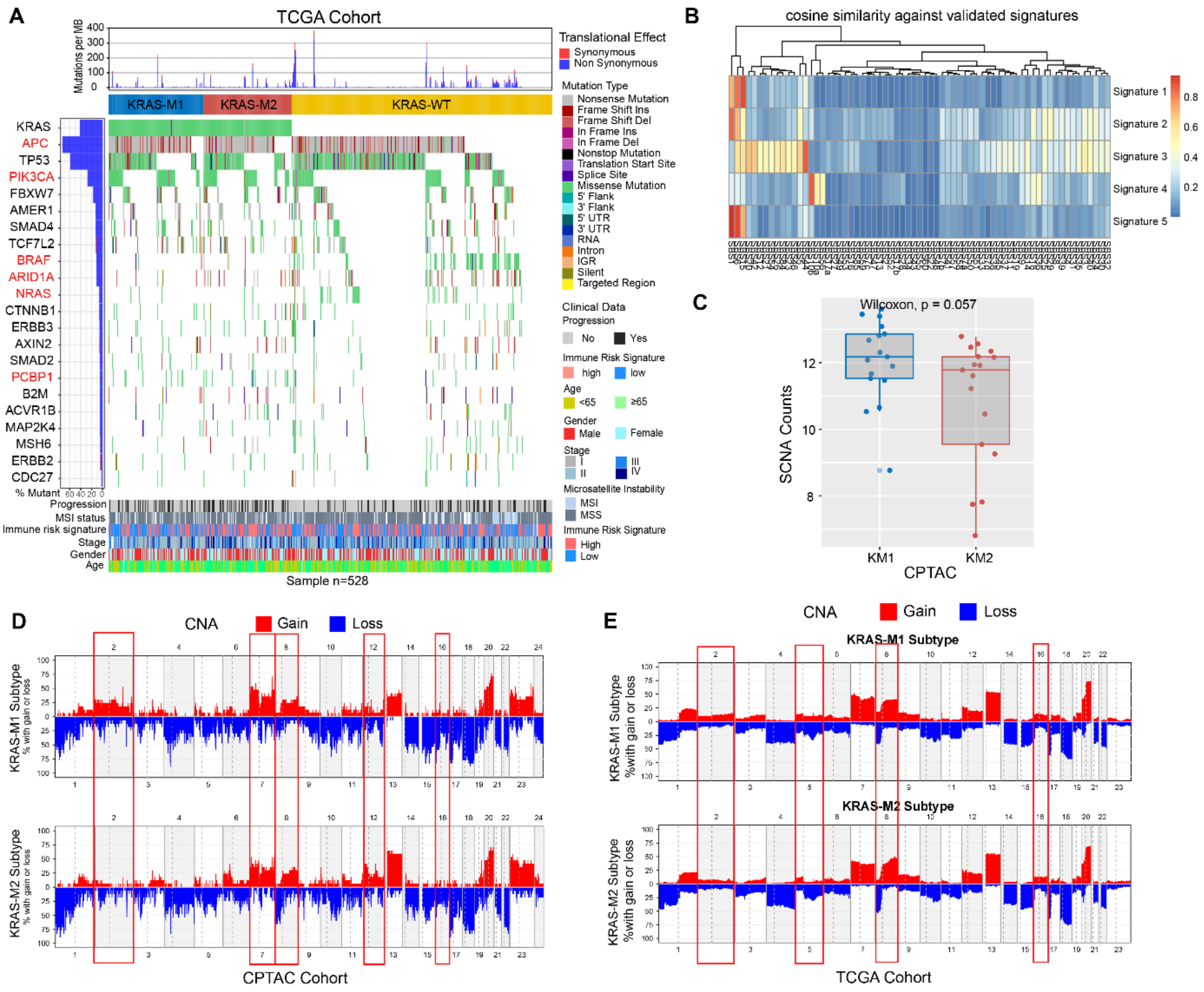

Figure S5. Tumor genomic alterations of KRAS-Mut colorectal cancer.

(A) Mutational landscape of SMGs in CPTAC stratified by KRAS-WT, KM1 and KM2 groups. Individual patients were represented in each column. The upper barplot showed TML, the right bar plot showed the mutation frequency of each gene in separate groups. Age, stage,

gender, MSI status, immune risk signature, and progression were shown as patient annotations. (B) Cosine similarity analysis of extracted mutational signatures against the 67 identified SBS signatures in Catalogue of Somatic Mutations in Cancer (COSMIC, v3) with heatmap illustration. (C) Relative distribution of arm level somatic copy number alternation in KM1 versus KM2 in CPTAC cohort. (D-E) Relative distribution of arm level somatic copy number alternation in KM1 versus KM2 in CPTAC cohort and TCGA cohort.

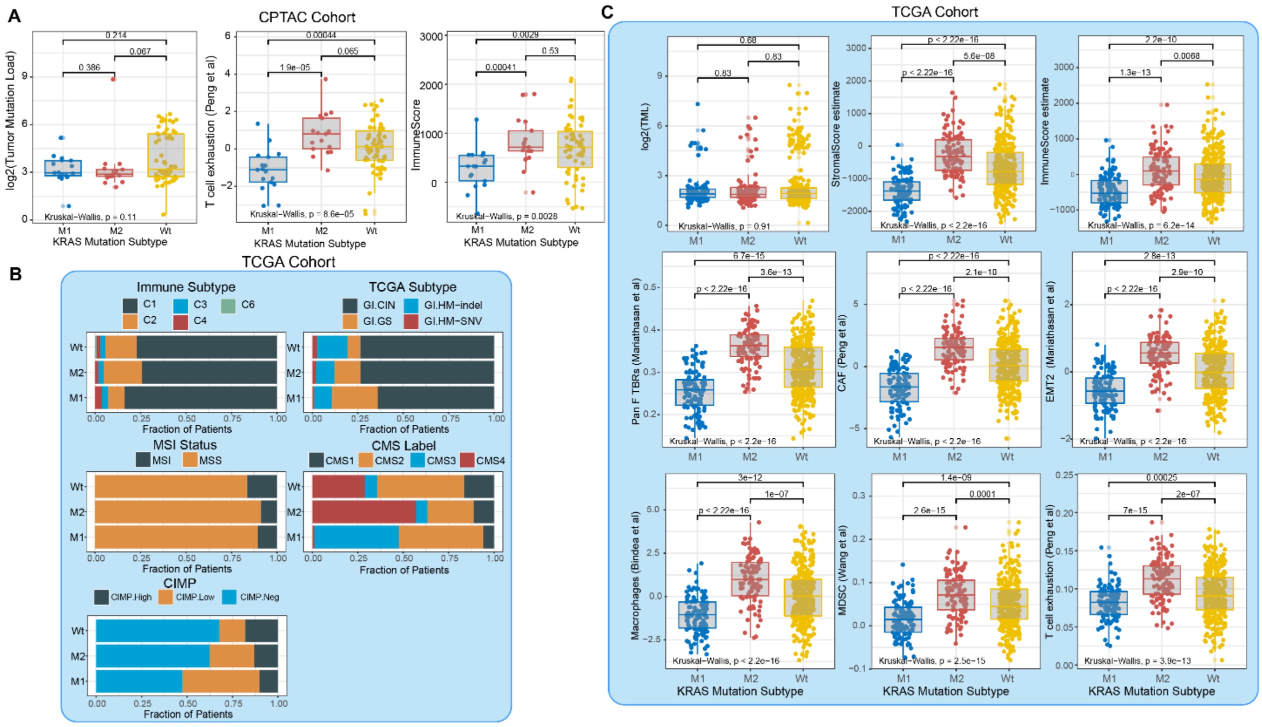

Figure S6. Molecular subtype and tumorigenic related signatures within KRAS-Mut subtypes in TCGA cohort.

(A) Differences in Tumor Mutation Load, T cell exhaustion, and Immune score among KM1, KM2 and WT groups in CPTAC cohort. (B) The proportion of TCGA immune subtypes, TCGA integrated subtype, MSI status, CMS label subtype, and CIMP status among KRAS-WT, KM1 and KM2 groups. (C) Differences in oncogenic-related signatures (TML,

stromal score, immune score, Pan F TBRs, CAF, EMT2, Macrophages, MDSC, and T cell exhaustion) among KRAS-WT, KM1 and KM2 groups in TCGA cohort.

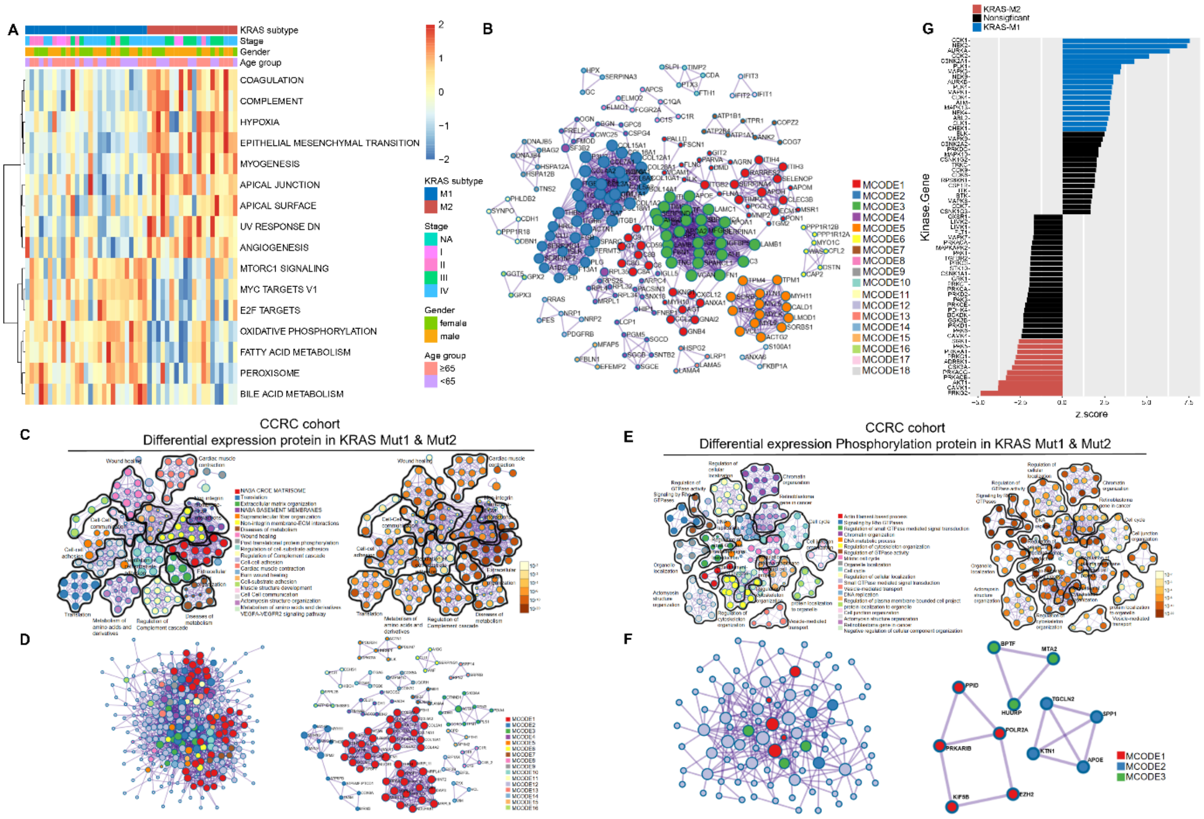

Figure S7. Proteomics and Phosphoproteomics characterzation of KRAS-Mut subgroups in CCRC cohort

(A) Heatmap shows the representative molecular pathways on proteomics among KM1 and KM2 groups in CCRC cohort. (B) Protein-protein interaction enrichment analysis by metascape analysis were performed to validate the relationship of differential expression protein in KM1 and KM2 tumors subset. Cluster MCODE1 included the proteins ITGB2, ECM1. APOH, et.al, which are associated with Platelet degranulation (R-HSA-114608); Complement and coagulation cascades (hsa04610); Platelet activation, signaling and aggregation (R-HSA-76002). Cluster MCODE2 included COL15A1, COL12A1, COL1A2, et.al, which are associated with the Extracellular matrix organization (R-HSA-1474244);

Integrin cell surface interactions (R-HSA-216083); NABA COLLAGENS (M3005). The remaining cluster MCODE were related to Extracellular matrix organization (R-HSA-1474244); NABA CORE MATRISOME (M5884), et.al. Colors represent the different MCODE clusters. PPI, Protein-protein interaction; MCODE, Molecular Complex Detection. (C-D) The proteomics and phosphoproteome data in CCRC cohort were curated and subjected to metascape analysis and found similar results to CPTAC cohort. According to the MCODE method, 16 sub-clusters of proteins were identified; proteins in each cluster shared the same GO terms and KEGG pathways. Cluster MCODE1 included the proteins COL4A2, COL6A2, COL1A1, et.al, which are associated with Collagen biosynthesis and modifying enzymes (R-HSA-1650814); post-translational protein phosphorylation (R-HSA-8957275); NABA CORE MATRISOME (M5884). The remaining cluster MCODE were related to Extracellular matrix organization (R-HSA-1474244); NABA CORE MATRISOME (M5884), et.al. (E-F) The proteomics and phosphoproteome data in CCRC cohort were curated and subjected to metascape analysis and found similar results to CPTAC cohort. According to the MCODE method, 3 sub-clusters of proteins were identified; proteins in each cluster shared the same GO terms and KEGG pathways. Cluster MCODE1 included the proteins KIF5B, EZH2, POLR2A, PPID and PPKARIB, et.al, which are associated with post-translational protein phosphorylation (R-HSA-8957275); Regulation of Insulin-like Growth Factor (IGF) transport and uptake by Insulin-like Growth Factor Binding Proteins (IGFBPs) (R-HSA-381426); chromatin organization (GO:0006325). The remaining cluster MCODE were related to chromatin organization (GO:0006325); chromatin assembly or

disassembly (GO:0006333); DNA metabolic process (GO:0006259). Colors represent the different MCODE clusters. PPI, Protein-protein interaction; MCODE, Molecular Complex Detection. (G) Enriched kinases in KM1 and KM2 subsets using KSEA with a significance of P < 0.05 in CCRC cohort. Red bars represent positively enriched; purple bars represent negatively enriched.

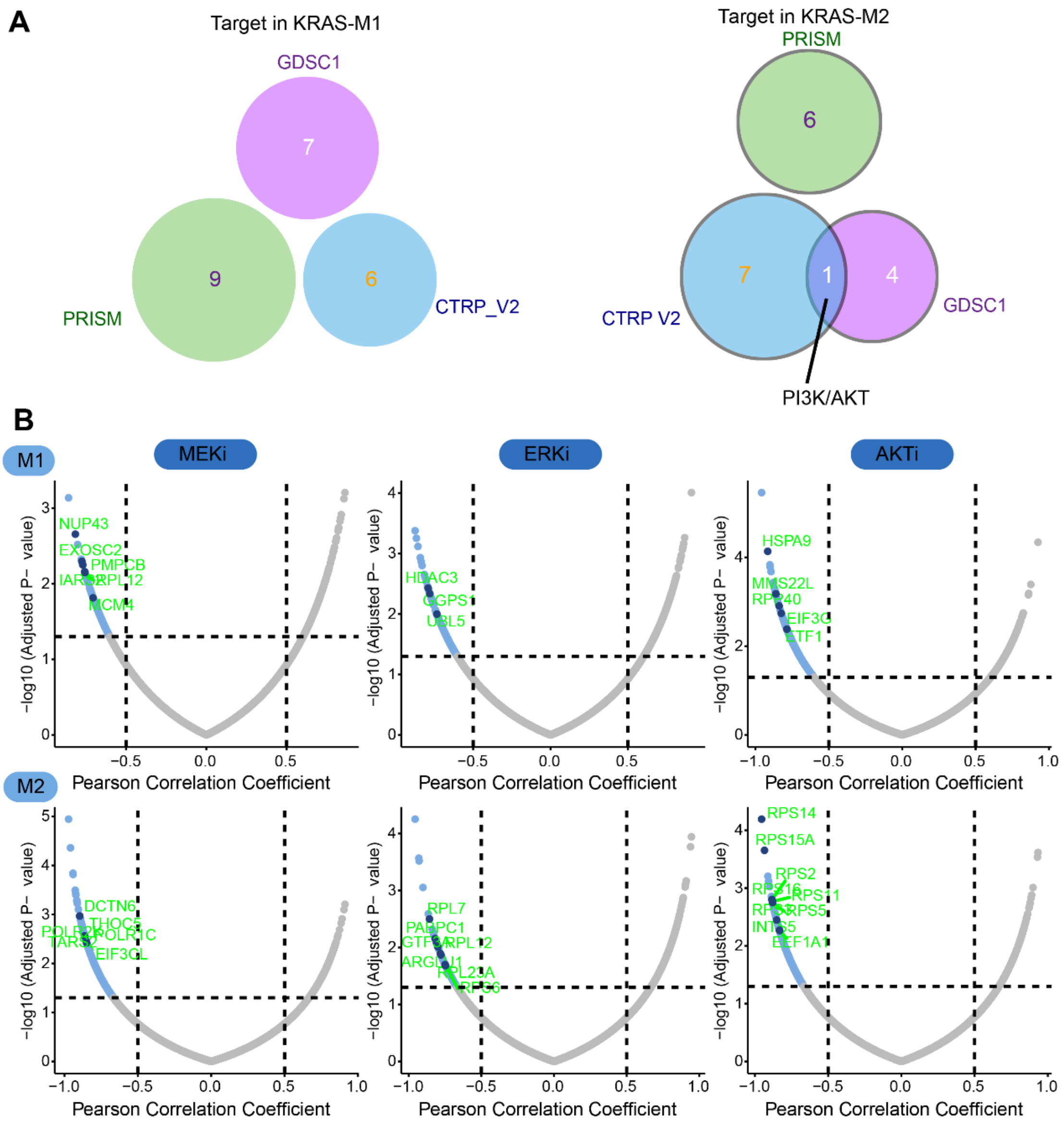

Figure S8. Molecular targets in CCLE.

(A) Overlapped molecular targets among the three drug sensitivity database in KM1 and KM2 subtype. (B) Correlations between the sensitivity of MEKi/ERKi/AKTi and transcriptomic

levels in each KRAS-Mut subset. Genes with Pearson correlation coefficient less than -0.5 and FDR < 0.05 was colored by blue and representative gene was marked by darkblue dot.

Table S1. Summary of included clinical cohorts.

Table S2. Clinical characteristics of included cohorts.

Table S3. Detailed clinical annotation of TCGA cohort.

Table S4. Comparison of RNA, protein, and phosphorylation site abundance changes between KRAS-Mut and WT tumors.

Table S5. Detailed clinical annotation of CPTAC cohort.

Table S6. Differential phosphosites among KM1 and KM2 subtype.

Table S7. Kinase-substrate enrichment analyses (KSEA) in CPTAC and CCRC cohorts.

Table S8. Drug annotation of selected agents for KM1 and KM2 subtypes.
